# N-ethylmaleimide-sensitive factor elicits a neuroprotection against ischemic neuronal injury by restoring autophagic/lysosomal dysfunction

**DOI:** 10.1038/s41420-024-02144-7

**Published:** 2024-08-18

**Authors:** Miaomiao Qiu, Xiaoming Zhao, Tao Guo, Hongyun He, Yihao Deng

**Affiliations:** 1 School of Basic Medical Sciences, Kunming University of Science and Technology, Kunming, 650500 China; 2 Anning First People’s Hospital Affiliated to Kunming University of Science and Technology, Kunming, 650500 China

**Keywords:** Macroautophagy, Cell death in the nervous system

## Abstract

Autophagosome-lysosome fusion defects play a critical role in driving autolysosomal dysfunction, leading to autophagic/lysosomal impairment in neurons following ischemic stroke. However, the mechanisms hindering autophagosome-lysosome fusion remain unclear. Soluble N-ethylmaleimide-sensitive factor (NSF) is an essential ATPase to reactivate STX17 and VAMP8, which are the paired molecules to mediate fusion between autophagosomes and lysosomes. However, NSF is frequently inactivated to inhibit the reactivation of STX17 and VAMP8 in ischemic neurons. Herein, we investigated whether autophagosome-lysosome fusion could be facilitated to alleviate autophagic/lysosomal impairment in ischemic neurons by over-expressing NSF. Rat model of middle cerebral artery occlusion (MCAO) and HT22 neuron ischemia model of oxygen-glucose deprivation (OGD) were prepared, respectively. The results demonstrated that NSF activity was significantly suppressed, accompanied by reduced expressions of STX17 and VAMP8 in penumbral neurons 48 h post-MCAO and in HT22 neurons 2 h post-OGD. Moreover, the attenuated autolysosome formation accompanied by autophagic/lysosomal dysfunction was observed. Thereafter, NSF activity in HT22 neurons was altered by over-expression and siRNA knockdown, respectively. After transfection with recombinant NSF-overexpressing lentiviruses, both STX17 and VAMP8 expressions were concurrently elevated to boost autophagosome-lysosome fusion, as shown by enhanced immunofluorescence intensity co-staining with LC3 and LAMP-1. Consequently, the OGD-created autophagic/lysosomal dysfunction was prominently ameliorated, as reflected by augmented autolysosomal functions and decreased autophagic substrates. By contrast, NSF knockdown conversely aggravated the autophagic/lysosomal impairment, and thereby exacerbated neurological damage. Our study indicates that NSF over-expression induces neuroprotection against ischemic neuronal injury by restoring autophagic/lysosomal dysfunction via the facilitation of autophagosome-lysosome fusion.

**Figure:**
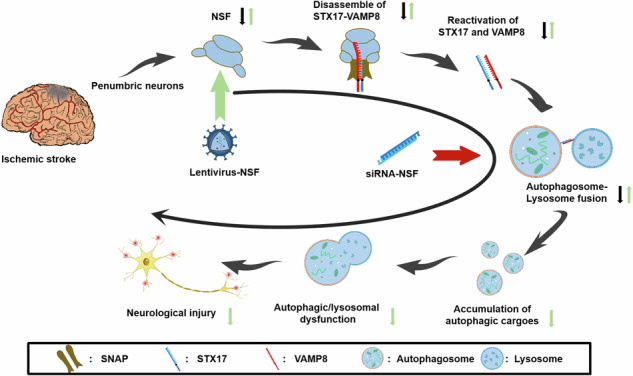
Over-expression of NSF promotes fusion by reactivating STX17 and VAMP8. Black arrows represent the pathological process after cerebral ischemia, green arrows represent the mechanism of remission after NSF over-expression, and red arrows represent the effect on the pathological process after NSF knockdown.

Over-expression of NSF promotes fusion by reactivating STX17 and VAMP8. Black arrows represent the pathological process after cerebral ischemia, green arrows represent the mechanism of remission after NSF over-expression, and red arrows represent the effect on the pathological process after NSF knockdown.

## Introduction

Cerebral stroke is a severe cerebrovascular disease, resulting in millions of deaths and disabilities annually worldwide [1]. Despite intensive investigation into the pathological mechanisms of cerebral ischemia in recent decades, effective treatment options remain limited [2]. The recombinant tissue plasminogen activator (rtPA) is an effective agent for thrombolytic therapy, but its therapeutic effectiveness is sharply abated beyond 4.5 h post-stroke [3]. Mechanical thrombectomy is another approach to remove cerebrovascular occlusion, but it may induce more severe ischemia/reperfusion injury due to the abrupt restoration of blood flow [4]. Thus, exploring the underlying pathogenesis of cerebral stroke remains a fundamental route to uncover novel therapies.

Autophagy was extensively participated in the pathophysiological processes of ischemic stroke [5]. An integral autophagy contained a series of consecutive processes: autophagy activation, autophagosome maturation, autolysosome formation by fusion of autophagosomes with lysosomes, and degradation of autophagic materials within autolysosomes. A sequential state from autophagy initiation to lysosomal degradation was termed autophagic/lysosomal signaling pathway, as well as autophagic flux [6]. Our previous study revealed that massive autophagic substrates were accumulated within neurons, leading to autophagic/lysosomal dysfunction after ischemic stroke [7]. This autophagic accumulation may result from either excessively generated autophagic cargoes or lysosomal inefficiency [8]. To distinguish what drove the impairment of autophagic flux, we intervened in autophagic activity and lysosomal capacity, separately. We found that the cerebral ischemia-created autophagic/lysosomal dysfunction could be greatly restored by augmenting lysosomal functions, but not by inhibiting autophagic activity [9]. This indicated that lysosomal inefficiency is a major pathogenesis to drive the impairment of autophagic flux. Moreover, we found that the fluorescence intensity of LC3 (an autolysosomal marker) and LAMP-1 (lysosome-associated membrane protein 1, a lysosomal indicator) co-labeling within the penumbral neurons were significantly reduced, implying that the fusion between autophagosomes and lysosomes was hampered [8]. Thus, we hypothesized that the deficiency of autolysosome formation might be an essential pathogeny of autophagic/lysosomal dysfunction in neurons after ischemic stroke [10].

The fusion between autophagosomes and lysosomes was mediated by the endocellular membrane-membrane fusion machinery, involving three core elements: N-ethyl-maleimide sensitive factor (NSF), soluble NSF attachment protein (SNAP), and soluble NSF attachment protein receptors (SNAREs) [11]. SNAREs were the proteins that directly mediated the membrane-membrane fusion [12]. After fusion, SNAREs formed complexes that were retained on fusion membranes with inactive states [13]. They must be dissociated by NSF to become individual active conformations for the next cycle of membrane fusion [14]. During reactivation of SNAREs, SNAP played an adapter role by attaching NSF to SNAREs [15]. It was vital that NSF was a unique ATPase to reactivate SNAREs [16]. However, recent investigations demonstrated that NSF was drastically inhibited in ischemic neurons [17]. Thus, we assumed that the reactivation of SNAREs might be attenuated by NSF inactivation to interrupt the fusion between autophagosomes and lysosomes [18], leading to reduction of autolysosome formation. This was likely the pathogenesis of autophagic/lysosomal dysfunction in neurons after ischemic stroke.

Syntaxin-17 (STX17) was a SNARE protein mainly expressed on autophagosomes [19], while vesicle-associated membrane protein 8 (VAMP8) was localized on lysosomes [20]. They were the paired SNAREs for the mediation of fusion between autophagosomes and lysosomes [21]. After fusion, STX17-VAMP8 complexes were formed to reside on the fusion membranes of autolysosomes, and were required to be dissociated by NSF to become individual active conformations. However, studies demonstrated that NSF activity was greatly inhibited, coupling with reduced expressions of STX17 and VAMP8 in ischemic neurons [22]. This indicated that the reactivation of STX17 and VAMP8 was suppressed by NSF inactivation under ischemic conditions. Therefore, this study aims to investigate whether the reactivation of STX17 and VAMP8 could be promoted by NSF over-expression to facilitate the fusion of autophagosomes with lysosomes, which restored autophagic/lysosomal dysfunction in ischemic neurons.

## Results

### Ischemia-inactivated NSF markedly inhibited reactivation of STX17 and VAMP8 in penumbral neurons

**Fig. 1:**
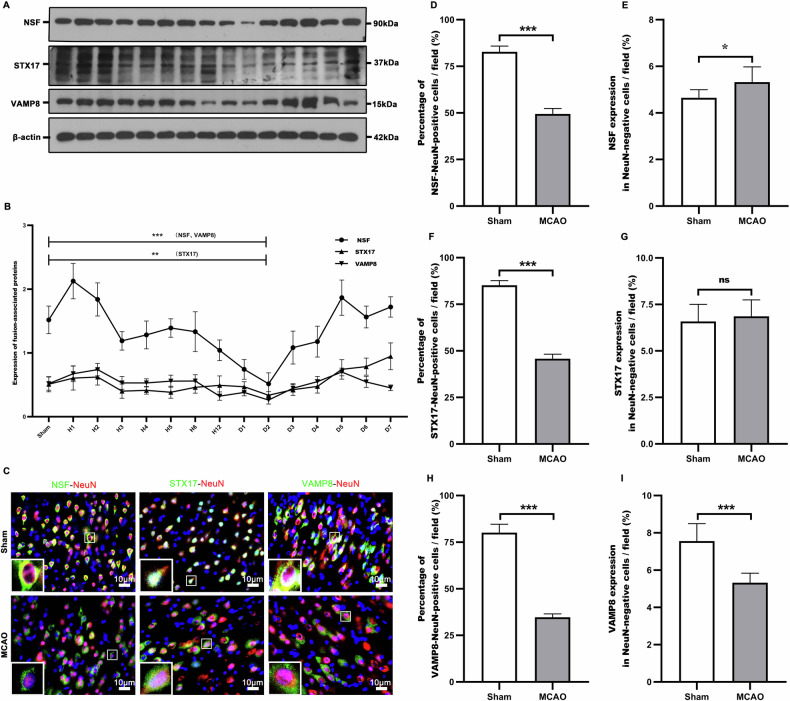
The correlative changes between NSF activity and expressions of STX17 and VAMP8 were revealed after ischemia. Western blot (**A**, **B**) showed that NSF activity was drastically inhibited, while the expressions of STX17 and VAMP8 was reduced in penumbral areas 48 h after MCAO. Immunofluorescence (**C**) showed that the inactivated NSF (**D**) and decreased expressions of STX17 (**F**) were predominantly occurred in neurons at the penumbra (**E**, **G**). The expression of VAMP8 (**H**, **I**) was also significantly reduced. *n* = 6. *p < 0.05, **P < 0.01, ***p < 0.001; ns indicates that the data are not statistically different.

The relevant alterations between NSF activity and expressions of STX17 and VAMP8 were investigated after ischemia. Western blot (Fig. 1A, B) demonstrated that NSF was significantly inactivated, coupling with decreased expressions of STX17 and VAMP8 in penumbral tissues 48 h after MCAO. Immunofluorescence (Fig. 1C) showed that the inhibited NSF (Fig. 1D) and reduced expressions of STX17 (Fig. 1F) were predominantly displayed in neurons at the penumbra (Fig. 1E, G), which also had significantly reduced VAMP8 levels (Fig. 1H, I). These data suggested that NSF inactivation greatly suppressed reactivation of STX17 and VAMP8 in ischemic neurons.

### The autophagic/lysosomal dysfunction might be driven by fusion interruption between autophagosomes and lysosomes after MCAO

**Fig. 2:**
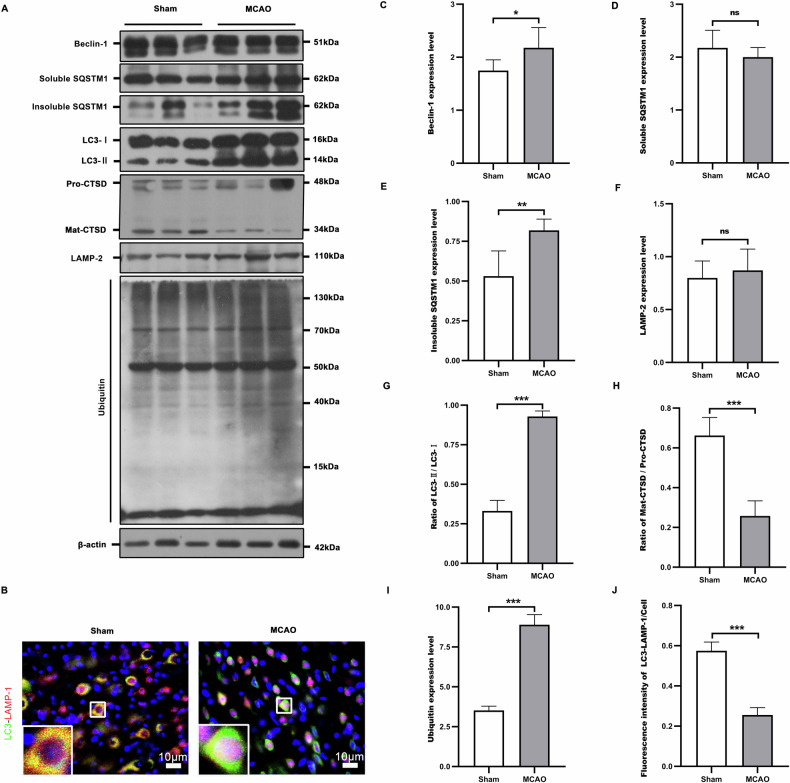
Disrupted fusion of autophagosomes with lysosomes might drive the autophagic/lysosomal dysfunction at the penumbra after MCAO. The penumbra tissues from Sham and 48 h after MCAO were analyzed by Western blot for autophagy flux related protein (**A**). The corresponding statistical graphs (**C**–**I**) were drawn according to the results. An impairment of autophagic flux was created, as shown by the increased autophagic substrates (**E**, **G**, **I**) and attenuated lysosomal capacity (**H**) 48 h after cerebral ischemia. Meanwhile, LC3-LAMP-1 immunofluorescence assessment was performed to determine autophagosome and lysosome fusion (**B**). Statistical graphs were generated based on the results (**J**). LC3-LAMP-1 co-labeling fluorescence intensity was significantly lower in the MCAO group compared with the Sham group. *n* = 6, *p < 0.05, **p < 0.01, ***p < 0.001, ns indicates that the data are not statistically different.

The penumbral tissues were obtained 48 h after MCAO. Western blot (Fig. 2A) demonstrated that cerebral ischemia created an autophagic/lysosomal dysfunction, as reflected by increased autophagic substrates of LC3-II (Fig. 2G), ubiquitinated (Fig. 2I) proteins and insoluble p62 (Fig. 2E), accompanied by decreased lysosomal CTSD (Fig. 2H). Immunofluorescence (Fig. 2B) showed that the intensity of fluorescence co-labeled with LC3-LAMP-1(Fig. 2J) in the penumbral neurons were significantly lower in the MCAO group compared with the sham group, suggesting that a fusion barrier was created between autophagosomes and lysosomes. Thus, we inferred that the impaired autophagic flux was likely driven by the interruption of autolysosome formation after ischemic stroke.

### NSF overexpression noticeably facilitated expression of STX17 and VAMP8 in ischemic neurons

**Fig. 3:**
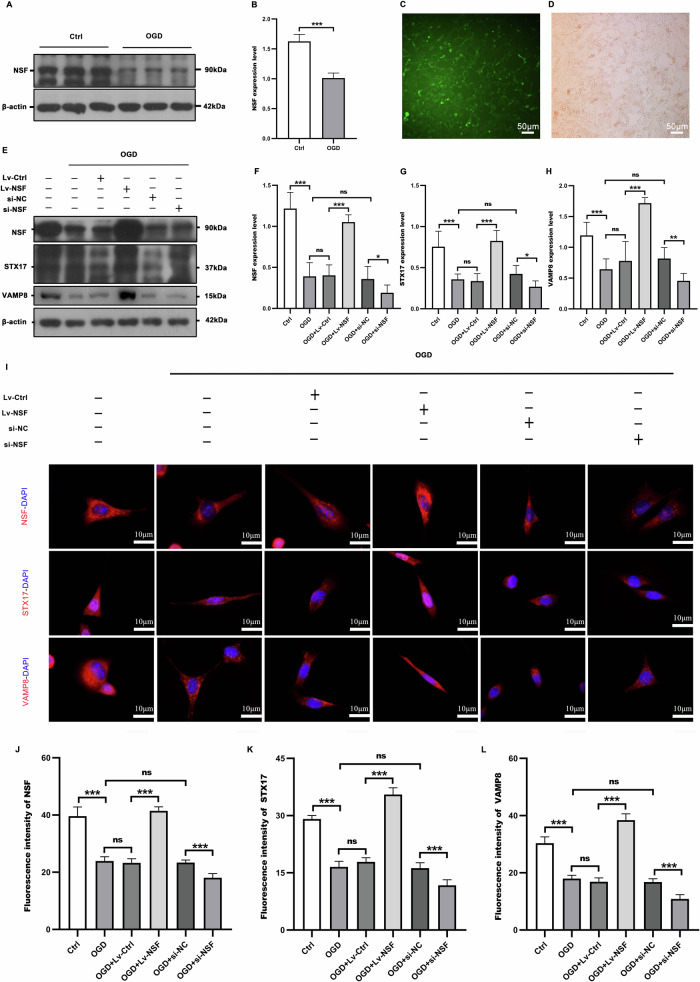
Over-expression NSF promotes the expression of STX17 and VAMP8 after OGD. NSF was significantly inactivated 2 h after OGD (**A**, **B**). NSF activity in OGD HT22 neurons was altered by the recombinant NSF-expressed lentiviruses and siRNA knockdown, respectively. It was monoclonal screened for a week, microscopic GFP fluorescence showed NSF over-expression (**C**) and the morphology was well (**D**). Western blot (**E**) and immunofluorescence (**I**) detection of NSF, STX17, and VAMP8 expression levels in HT22 neurons, and the corresponding statistical graphs were plotted (**F**–**H**, **J**–**L**). NSF over-expression (**F**, **J**) significantly promoted the expression of STX17 (**G**, **K**) and VAMP8 (**H**, **L**). *n* = 6, *p < 0.05, **p < 0.01, ***p < 0.001, ns indicates that the data are not statistically different.

NSF activity in OGD HT22 neurons was altered by the recombinant NSF-expressed lentiviruses and siRNA knockdown, respectively. Western blot (Fig. 3A, B) showed that NSF activity was significantly suppressed 2 h after OGD. After 8 days of co-culture, HT22 neurons could be effectively transfected by the recombinant NSF-expressed lentiviruses, as confirmed by approximately 90% of the GFP-labeled cells (Fig. 3C, D) and elevated NSF expression (Fig. 3E). Moreover, the reinforced NSF activity greatly promoted the expression of STX17 (Fig. 3F, G) and VAMP8 (Fig. 3F, H) in OGD+Lv-NSF group, compared with those in OGD+Lv-Ctrl group. Furthermore, immunofluorescence also validated that NSF over-expression significantly promoted the expression of STX17 (Fig. 3I, K) and VAMP8 ((Fig. 3I, L), as reflected by enhanced fluorescence intensity in OGD+Lv-NSF group, compared with those in OGD+Lv-Ctrl group. Compared with OGD+si-NC, NSF knockdown significantly inhibited the activity of NSF while further suppressing the expression of STX17 and VAMP8 (Fig. 3E-L).

### NSF over-expression greatly promoted reactivation of STX17 and VAMP8 in ischemic neurons

**Fig. 4:**
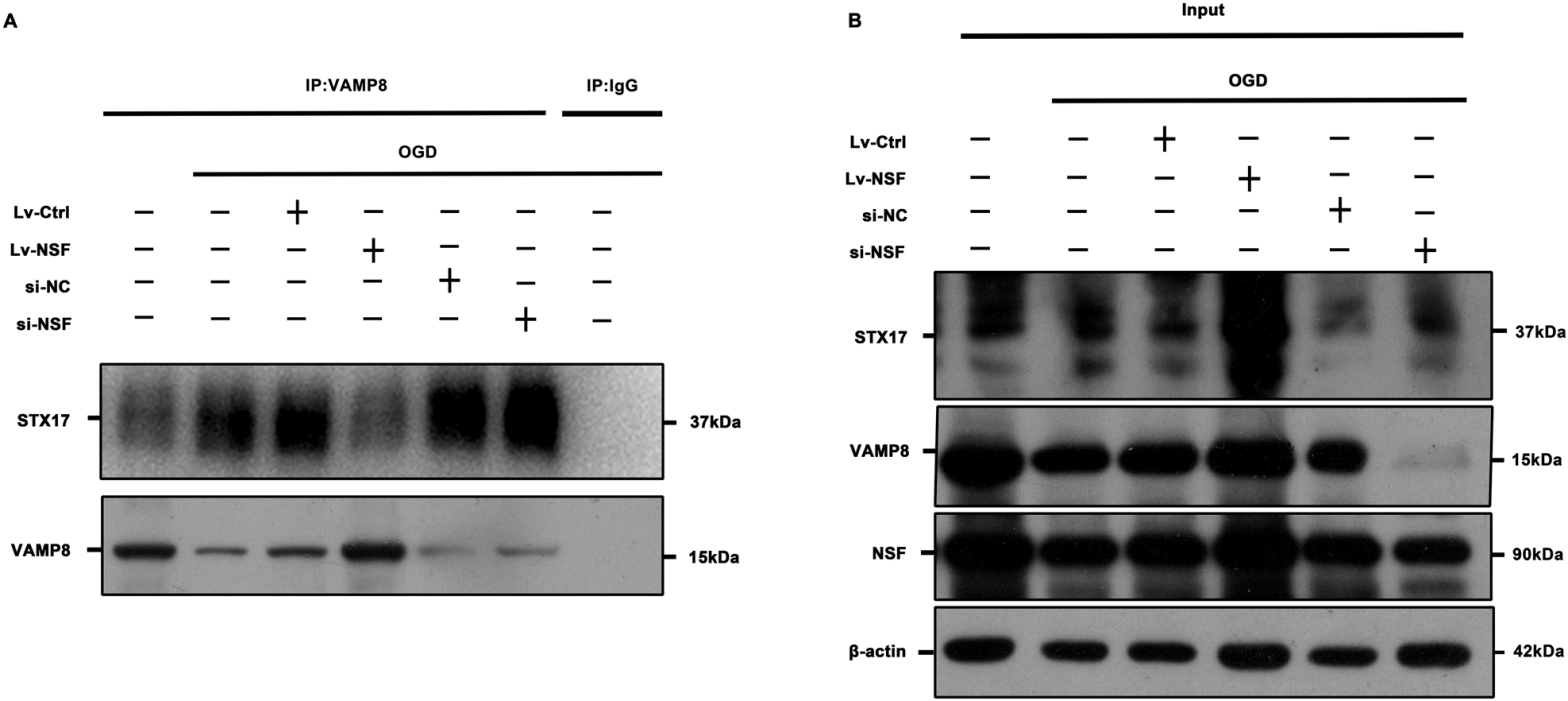
Over-expressing NSF prominently promoted reactivation of STX17 and VAMP8 in OGD HT22 cells. Detection of the relationship between NSF and STX17 and VAMP8 proteins and changes after OGD by Co-immunoprecipitation assay (**A**, **B**). NSF over-expression markedly reduced STX17-VAMP8 and promoted the reactivation of STX17 and VAMP8 (**A**). Consequently, the expressions of STX17 and VAMP8 were markedly promoted (**B**). IP: immunoprecipitated with VAMP8 antibodies; IgG: Anti-rabbit IgG; Input: whole cell lysate. *n* = 6, *p < 0.05, **p < 0.01, ***p < 0.001, ns indicates that the data are not statistically different.

Co-immunoprecipitation (Fig. 4A, B) was performed to verify the effect of NSF elevation on reactivations of STX17 and VAMP8 in HT22 neurons 2 h after OGD. The results demonstrated that STX17-VAMP8 complexes were markedly reduced in OGD+Lv-NSF group, compared with those in OGD+Lv-Ctrl group. Moreover, both STX17 and VAMP8 (Fig. 4B) expressions were significantly promoted by NSF over-expression. In contrast, NSF siRNA knockdown instead increased the STX17-VAMP8 complex (Fig. 4A), thereby attenuating STX17 and VAMP8 expression (Fig. 4B), compared with the OGD+si-NC group. In conclusion, these data suggest that NSF overexpression in OGD HT22 neurons effectively promotes the reactivation of STX17 and VAMP8.

### NSF elevation drastically facilitated autophagosome-lysosome fusion in ischemic neurons

**Fig. 5:**
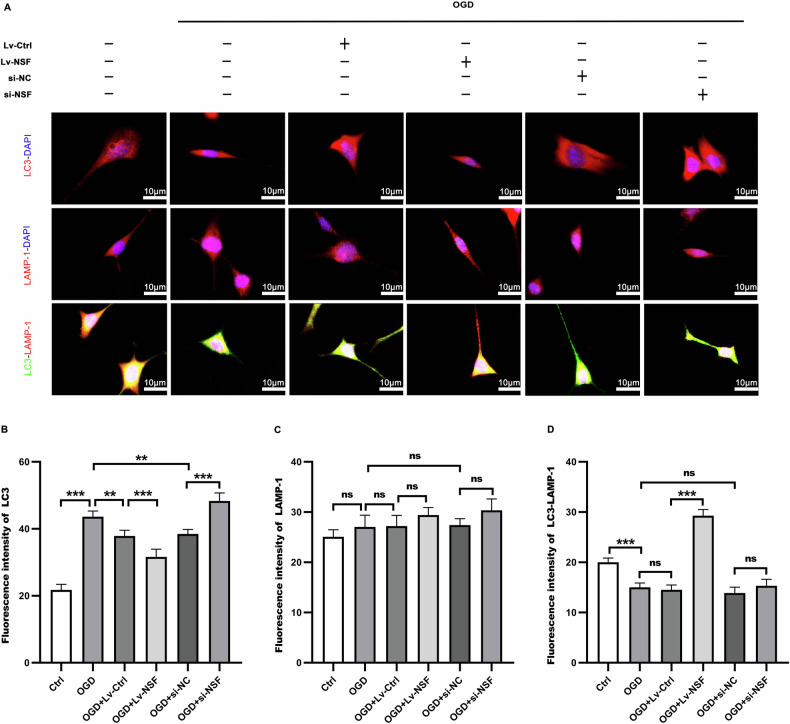
NSF over-expression promotes autophagosome and lysosome fusion after OGD. The autophagosome and lysosome fusion in HT22 neurons were detected by immunofluorescence of LC3-Neun, LAMP-1-Neun, and LC3-LAMP-1 (**A**), and the corresponding statistical graphs were plotted according to the results (**B**–**D**). There was no statistically significant difference in the fluorescence intensity of LAMP-1 (**C**) among groups. The individual expression of LC3 (**B**) was prominently promoted by OGD, but the fluorescence intensity co-stained with LC3-LAMP-1 (**D**) were prominently lower than that individually labeled with LC3 (**B**) as well as LAMP-1 (**C**). After NSF over-expression, the co-staining of LC3-LAMP-1 (**D**) was greatly enhanced. *n* = 6, *p < 0.05, **p < 0.01, ***p < 0.001, ns indicates that the data are not statistically different.

The fusion between autophagosomes and lysosomes in HT22 neurons was evaluated 2 h after OGD. Immunofluorescence (Fig. 5A) showed that the fluorescence intensity of LAMP-1 was not statistically different (Fig. 5C) among groups, and LC3 (Fig. 5B) expression was even increased in OGD group, compared with those in sham group. However, the fluorescence intensity co-stained with LC3-LAMP-1 (Fig. 5D) was prominently lower than that individually labeled with LC3 as well as LAMP-1. This suggested that the fusion between autophagosomes and lysosomes was hampered by OGD. After transfection with the NSF-expressed lentiviruses, the fluorescence intensity co-staining of LC3 with LAMP-1 (Fig. 5D) was significantly enhanced in OGD+Lv-NSF group compared with that in OGD+Lv-Ctrl group. In contrast, siRNA-mediated knockdown of NSF instead further reduced LC3-LAMP-1 co-expression compared with the OGD+si-NC group (Fig. 5D). These data indicated that NSF elevation effectively boosted autophagosome-lysosome fusion in OGD HT22 neurons.

### NSF up-regulation significantly restored the autophagic/lysosomal dysfunction by augmenting autolysosomal capacity in OGD HT22 neurons

**Fig. 6:**
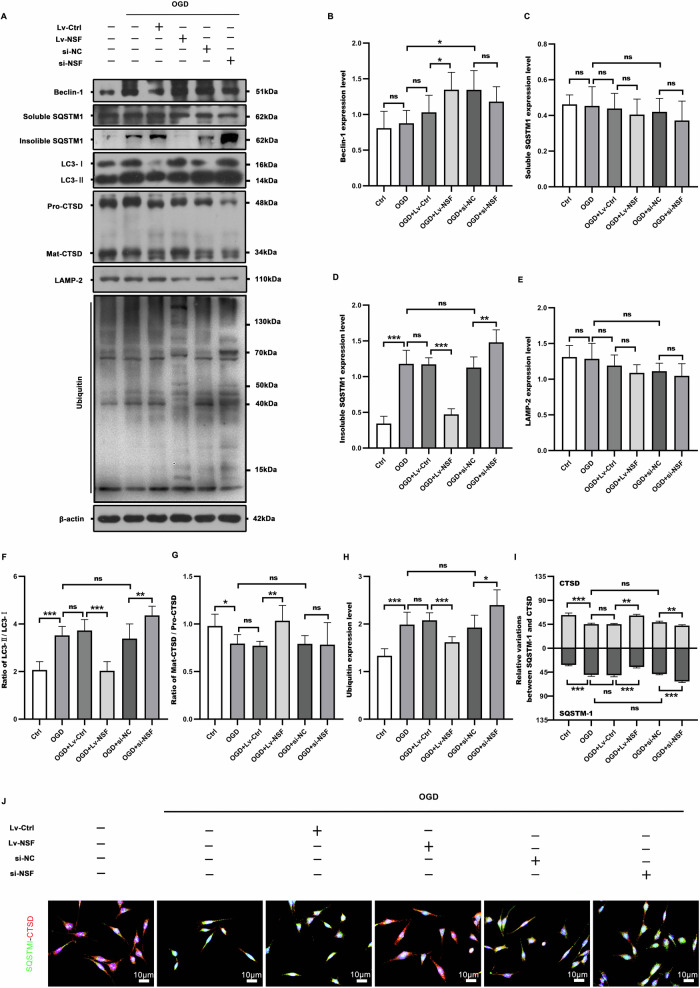
NSF elevation greatly restored the autophagic/lysosomal dysfunction in OGD HT22 neurons. Expression levels of autophagic flux related proteins in HT22 neurons after OGD were detected by western blot analysis (**A**) and the corresponding statistical graphs (**B**–**H**) were plotted according to the results. The OGD-created autophagic/lysosomal dysfunction could be significantly alleviated by NSF over-expression, as indicated by reduced autophagic substrates (**D**, **F**, **H**) and augmented autolysosomal function (**G**). The co-staining of SOSTM-1-CTSD (**J**) statistics (**I**) showed that NSF over-expression promoting CTSD expression was accompanied by a significant reduction of SQSTM-1. *n* = 6, *p < 0.05, **p < 0.01, ***p < 0.001, ns indicates that the data are not statistically different.

To investigate the effect of NSF-facilitated autolysosome formation on autophagic flux, the HT22 neurons were obtained 2 h after OGD. The western blot (Fig. 6A) results showed that OGD drove an autophagic/lysosomal dysfunction, as indicated by increased autophagic substrates (Fig. 6D, F, H) and weakened autolysosomal functions (Fig. 6G) in OGD group, compared with those in sham group. After infection with the recombinant lentiviruses over-expressing NSF, the OGD-created impairment of autophagic flux was greatly restored, as reflected by decreased autophagic cargoes of LC3-II (Fig. 6F), insoluble p62 (Fig. 6D) and ubiquitinated (Fig. 6H) proteins, accompanied by increased CTSD (Fig. 6G). Moreover, the lysosomal capacity was significantly augmented by NSF up-regulation, as confirmed by the significant elevation of CTSD (Fig. 6H, J) accompanied by a significant decrease in SQSTM-1 (Fig. 6H, J). In contrast, siRNA-mediated knockdown of NSF further exacerbated autophagic/lysosomal dysfunction (Fig. 6A–J) in OGD HT22 neurons compared to OGD+si-NC.

### NSF over-expression prominently alleviated ischemic neuronal injury

**Fig. 7:**
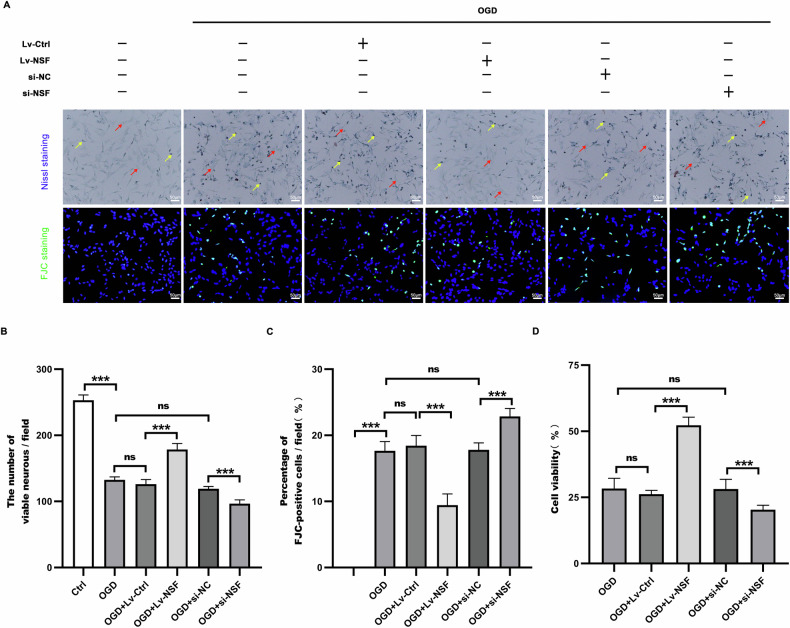
Over-expression of NSF effectively mitigated ischemic neuronal injury. Compared with those in OGD +Lv-ctrl group, the improved cell viability (**D**), promoted the number of Nissl bodies (**A**, **B**) and decreased FJC-positive cells (**A**, **C**) were detected in OGD + NSF group. This suggests that cellular protection against ischemia was induced by NSF over-expression. The yellow arrows indicated viable neurons, and the red arrows showed pyknotic neurons. *n* = 6, *p < 0.05, **p < 0.01, ***p < 0.001, ns indicates that the data are not statistically different.

Cell viability, Nissl staining and FJC staining (Fig. 7A) were performed to evaluate neuronal survival 2 h after OGD. NSF over-expression significantly alleviated the ischemic injury, as indicated by improved cell viability (Fig. 7D) and Nissl bodies (Fig. 7B), coupling with reduced FJC-positive cells (Fig. 7C). Conversely, siRNA-mediated NSF knockdown oppositely exacerbated the ischemic neuronal injury (Fig. 7A–D), compared with that in OGD+si-NC group.

## Discussion

Lysosomal inefficiency was an important pathogenesis of autophagic/lysosomal dysfunction in neurons after ischemic stroke [23]. However, lysosomal storage was conversely observed in ischemic neurons [24], implying that lysosomal deficiency was unlikely caused by the insufficiency of lysosomal biosynthesis. Our previous study demonstrated that that the fluorescence intensity of LC3 and LAMP-1 co-staining were significantly reduced in the penumbral cells [8], indicating the fusion between autophagosomes and lysosomes was interrupted after cerebral ischemia [25]. Thus, we hypothesized that deficiency of autolysosome formation might be a critical pathogenesis of lysosomal inefficiency, which consequently drove the impairment of autophagic flux in neurons after cerebral stroke [26]. STX17 and VAMP8 were the paired molecules to mediate the autophagosomes-lysosomes fusion [27]. However, studies showed that both STX17 and VAMP8 expressions were markedly reduced [22], accompanied by NSF inhibition in ischemic neurons [17]. Thus, we believed that the insufficiency of autolysosome formation was likely caused by decreased reactivation of STX17 and VAMP8, due to NSF inactivation. Therefore, the present study was to investigate whether the autophagic/lysosomal dysfunction could be restored to alleviate ischemic neuronal injury by over-expressing NSF.

We first investigated the correlation variations between NSF activity and the expressions of STX17 and VAMP8 after the ischemia. The results demonstrated that NSF was prominently inhibited, coupling with decreased expressions of STX17 and VAMP8 in penumbral neurons 48 h after ischemic stroke, as well as in HT22 neurons 2 h after OGD. This suggested that cerebral ischemia-inactivated NSF greatly suppressed reactivation of STX17 and VAMP8 [28]. Further investigation in OGD-treated HT22 neurons revealed that transfection with recombinant NSF lentiviruses significantly increased NSF activity and promoted STX17 and VAMP8 expressions. Co-immunoprecipitation demonstrated a reduction in STX17-VAMP8 complexes in the OGD + NSF group compared to the OGD group, confirming that recombinant NSF reactivates STX17 and VAMP8. Furthermore, immunofluorescence showed that the fluorescence intensity co-staining with LC3 and LAMP-1 was greatly enhanced, indicating the fusion between autophagosomes and lysosomes was facilitated by NSF upregulation. Subsequently, the OGD-created autophagic/lysosomal dysfunction was greatly restored, as reflected by the augmented autolysosomal functions and decreased autophagic substrates in OGD+Lv-NSF group, compared with those in OGD group. Collectively, these results validated that NSF-boosted autolysosome formation could effectively alleviate the impairment of autophagic flux. Moreover, the ischemic injury was significantly mitigated, as indicated by the improved cell viability and neuronal survival. By contrast, NSF knockdown conversely aggravated the impairment of autophagic flux, due to interruption of autolysosome formation. Consequently, the neurological injury was alleviated in OGD+siRNA group, compared with that in OGD group. Accordingly, we concluded that NSF over-expression conferred a neuroprotection against ischemic neuronal injury by alleviating the impairment of autophagic flux via facilitation of autolysosome formation.

The neuroprotective mechanism of NSF elevation in alleviating ischemic neuronal injury was preliminarily elucidated by this study. However, the effect of NSF over-expression on facilitating fusion of autophagosomes with lysosomes should be further verified in vivo MCAO model. In fact, the recombinant adeno-associated viruses expressing NSF had been administrated by intracerebroventricular injection in rat MCAO model in our study. Unfortunately, the penumbral cells were inefficiently infected by the recombinant viruses, leading to the failure of NSF over-expression. Previous studies demonstrated that cerebral ischemia often triggered strong inflammatory responses [29]. Thus, we speculated that the recombinant NSF adeno-associated viruses might be largely eliminated by the inflammatory clearance, leading to inefficiency of the transfection. Therefore, the regulative effect of NSF over-expression on ameliorating autophagic flux was only elucidated in cell ischemia model of HT22 neurons. Nevertheless, our data supported that NSF over-expression contributed to neuroprotection against ischemic neuronal injury.

## Materials and methods

### Experimental animals

Adult pathogen-free male Sprague Dawley (weighing 250g-280g, weekly age 10-12 weeks) rats were purchased from Beijing Fu kang Bio-technology Co. Ltd (License No. SCXK (Beijing) 2019-0008), and kept under standard conditions with free access to water and food. All animal experiments were approved by the Animal Experimentation Committee of Kunming University of Science and Technology (Approval No. 5301002013655) and were fed under the provisions of the Laboratory Animal Welfare Guidelines. A total of 122 rats were recruited, of which 20 died during or after surgery (mortality rate of approximately 16.4%). A remaining 102 rats were included in the study, of which 90 were investigated for changes in NSF, STX17, and VAMP8 after surgery (*n* = 6, 15 time points) and 12 were used for immunofluorescence experiments (*n* = 6, 2 groups), all of which were randomly assigned to different experimental groups. This part of the experiment was conducted using a double-blind method.

### Preparation of the middle cerebral artery occlusion (MCAO) model

As described previously, middle cerebral artery occlusion surgery was conducted in rats [30]. Briefly, rats were anesthetized with 2% sodium pentobarbital (50 mg/kg; Sigma-Aldrich, St. Louis, MO, USA), the left common carotid artery, external carotid artery, and internal carotid artery were respectively isolated, and a nylon monofilament coated with a round silicone (Cinontech, Beijing, China) was then entered into the internal carotid artery through an incision on the external carotid artery, and advanced by about 2 cm for occlusion of the middle cerebral artery (MCAO). Monofilament was removed after 1.5 h MACO for reperfusion. During and after the surgery, rats were placed on an electric heating pad (Rainbow, TG104-X32, Chengdu, China) to maintain body temperature at 37 °C. The rats in sham group were accepted for all manipulations except for monofilament insertion.

### Experimental cell

Mouse hippocampal neuronal cells HT22 were purchased from Procell Life Technology Co, Ltd. (Wuhan, China) and cultured at 37 °C, 5% CO2, 95% air environment in high glucose DMEM medium (HyClone, UT, USA), containing 10% fetal bovine serum (Biological Industries, CT, USA), 100 U/ml streptomycin and 0.1 mg/ml penicillin mixture (Procell, Wuhan, China). The cells have recently undergone STR profiling as well as mycoplasma testing, both of which are fine. The cell experiment consisted of 6 experimental groups, and the cells used for each experiment in each experimental group were passed on from the same bottle of cells and cultured under the same culture conditions (*n* = 6).

### NSF-expressed lentivirus transfection and siRNA knockdown

Lentivirus-NSF(PGMLV-CMV-Mouse-Nsf-EF1-ZsGreen1-T2A-Puro,1×108TU/ml, gene ID 8195, 55799GU-LV) was recombined and provided by Jiman Biotechnology (Jiman Biotechnology Co. Ltd, Shanghai, China). The Lv-NSF was added into the HT22 culture with 0.5 of MOI, for transfection. After 48 h of co-culture, the medium was replaced and incubated with puromycin (1 μg/ml, Thermo Fisher Scientific, MA, USA) for a total of 7 days to sort monoclonal cells, stably expressing NSF. The transfection efficiency was verified by GFP immunofluorescence and western blot analysis. The siRNA-NSF (20μmol/L) was provided by Jiman Biotechnology Co. Ltd (Shanghai, China) and added into HT22 culture with a dose of 50nmol/L, using Lipofectamine 2000 (Thermo Fisher Scientific, MA, USA) as transfection reagent. After 6 h, medium was replaced and continuingly cultured for 48 h, the validity of knockdown was confirmed by western blot analysis.

### Preparation of oxygen glucose deprivation (OGD) in HT22 cells

Oxygen Glucose Deprivation model in HT22 cells was prepared as described previously [31]. In brief, firstly, the cells were washed with 1×PBS buffer and were cultured in serum-free and sugar-free DMEM medium (glucose deprivation) at 37 °C, 5% CO^2^, and 95% N^2^ environment (oxygen deprivation). After 1.5 h of OGD, cells were returned to the standard culture conditions, and the cell samples were collected after 2 h of continuing culture.

### Western blot

The total proteins of cells and brain tissues were extracted using a RIPA lysate (Beyotime Biotechnology, Shanghai, China), and insoluble sequestosome 1 (SQSTM1)/p62 from the lysate was extracted by an Inclusion Body Solubilizing Buffer Kit (Shanghai Sangong Biotechnology Co., Ltd., Shanghai, China). The total protein concentration was determined by a BCA Protein Assay Kit (Shanghai Beyotime Biotechnology Co., Ltd.). Proteins with different molecular weights were separated by SDS-PAGE (10%-12%) and electrotransferred onto PVDF membranes (Millipore, Billerica, MA, USA). The membranes were blocked for 2 h at room temperature using 10% skimmed milk (Beyotime Biotechnology, Shanghai, China). After washing, they were incubated with primary antibody overnight (4 °C), and then incubated with secondary antibody for 1.5 h at room temperature, and washed for 2 h. After that, it was immersed in the Immobilon Protein Immunoblotting HRP Substrate (Millipore, Billerica, MA, USA), and protein signals were detected using X-ray film. β-actin was measured as a normal control, and the density of the fluorescent signal of the protein bands was measured using Image J software. The antibodies used in this study were rabbit antibodies against rat NSF (1:1000, Abclonal Technology, Wuhan, China), STX17 (1:1000, Thermo Fisher Scientific, MA, USA), VAMP8 (1:10000, Abcam. Cambs, UK), LAMP-2 (1:2000, Sigma-Aldrich, St. Louis, MO, USA), Beclin 1 (1:2000, Thermo Fisher Scientific, MA, USA), SQSTM1 (1:5000, Abcam, Cambs, UK), LC3 (1:5000, Sigma-Aldrich, St. Louis, MO, USA), ubiquitin (1:1000, Proteintech, Wuhan, China), β-actin (1:10,000, Abclonal, Wuhan, China), and mouse antibody against rat CTSD (1:2000, Thermo Fisher Scientific, MA, USA). All secondary antibodies (1:20,000, Cell Signaling Technology, MA, USA) were directed against rabbit and mouse IgG.

### Immunofluorescence

Rats were deeply anesthetized and sequentially perfused with saline followed by 4% paraformaldehyde (Invitrogen, Carlsbad, CA, USA). After perfusion, the brains were quickly removed and immersed in 4% paraformaldehyde overnight fixation, then dehydrated in 30% sucrose (Solarbio, Beijing, China), and sliced into 20μm thickness of coronal slices with a freezing microtome (SLEE, Mainz, Germany). After washing, the sections were permeabilized with 0.2% Triton X-100 (Solarbio, Beijing, China) for 15 min, then blocked with 10% BSA (Beyotime Biotechnology, Shanghai, China) for 1 h. Then, the sections were incubated with primary antibody overnight (4 °C). After washing, the brain sections were incubated with secondary antibody at room temperature for 2 h under dark. After washing, the nuclei were stained with DAPI (1:1000, Cell Signaling Technology, Danvers, MA, USA) for 5 min and observed and photographed with a fluorescence microscope (Nikon Instruments Co., Ltd., Tokyo, Japan). Finally, the results were expressed as the percentage of positive cells expressed the results. The cells were fixed with 4% paraformaldehyde for 15 min, and then permeabilized with 0.2% Triton X-100 (Solarbio, Beijing, China) for 10 min, and the subsequent steps were the same as those for the animal sections. The results were expressed as fluorescence intensity. The primary antibodies used were as follows: rabbit antibodies against rat NSF (1:200, Abclonal Technology, Wuhan, China), STX17 (1:250, Thermo Fisher Scientific, MA, USA,), VAMP8 (1:300, Abcam, Cambs. UK), LC3 (1:400, Sigma-Aldrich, St. Louis, MO, USA), SQSTM1 (1:400, Abcam, Cambs, UK), NeuN (1:400, Abcam, Cambs, UK). Mouse antibodies against rat CTSD (1:400, Thermo Fisher Scientific, MA, USA), LAMP-1 (1:300, Abclonal, Wuhan, China), NeuN (1:400, Abcam, Cambs, UK). The secondary antibodies used were Alexa Fluor coupled secondary antibodies (1:800, Jackson ImmunoResearch, West Grove, PA, USA).

### Co-IP

Immunoprecipitation was performed by a kit (Protein A + G magnetic bead assay, Beyotime Biotechnology, Shanghai, China). Briefly, cell samples were fully lysed thoroughly on ice for 10 min using a lysate containing inhibitors. After centrifugation, the supernatant was collected and the total protein concentration was determined by a BCA protein assay kit. (Shanghai Beyotime Biotechnology Co., Ltd.). A portion of the supernatant was incubated with Protein A + G magnetic beads conjugated to normal IgG or VAMP8 (5 μg/ml, Abcam, Cambs, UK) at 4 °C overnight, washed and denatured. The interactions of the target proteins were verified by western blot analysis. The remaining supernatant served as input sample, used as positive control, and target proteins were analyzed by western blot after denaturation.

### Nissl staining

The cells were fixed with 4% paraformaldehyde for 15 min and washed. Then they stained with Nissl staining solution (Beyotime Biotechnology, Shanghai, China) at 37 °C for 10 min, washed twice with distilled water for a few seconds each time, and then immersed for 5 seconds in 95% ethanol, washed twice with 70% ethanol, directly observed by an optical microscope (Nikon Instruments Co., Ltd., Tokyo, Japan). The results were expressed as the number of Nissl bodies at high magnification (×200).

### Fluoro-Jade C staining

HT22 cells were fixed with 4% paraformaldehyde for 15 min and washed, and they were stained with Fluoro-Jade C (FJC) staining kit (Biosensis, TR-100-FJT, Thebarton, Australia). First, they were immersed in a mixture of NaOH and ethanol (1%/80%) for 5 min, then in 70% ethanol solution for 2 min. After washing, they were incubated with 0.06% potassium permanganate solution for 10 min to optimize the fluorescence background, washed with distilled water, and treated with FJC solution for 10 min in the absence of light. Finally, the nuclei were stained for 5 min with DAPI, washed, and placed under a fluorescence microscope (Nikon Instruments Co., Ltd., Tokyo, Japan). The results were expressed as the percentage of FJC-positive cells at high magnification (×200).

### Cell viability assay

HT22 cells were assayed for cell viability by CCK-8 kit (Beyotime Biotechnology, Shanghai, China), 10 μl of CCK-8 Solution was added to each well and incubated at 37 °C for 2 h. Absorbance values at 450 nm were measured using an enzyme marker. Cell viability (%) =[(A-C)/(B-C)] ×100% (A represents experimental group absorbance values, B represents control group absorbance values, and C represents medium alone absorbance values).

### Statistical analysis

All data were expressed as mean ± standard deviation (SEM) and all statistical analyses were performed using Graph Pad Prism 8.0 (San Diego, CA, USA). Protein blots were analyzed using ImageJ. When data were normally distributed, comparisons of data between groups were made using the paired t-test (Figs. 1D–I and 2), and one-way analysis of variance (ANOVA), with Wilcoxon’s rank-sum test used in cases of noncompliance. Values of *P* < 0.05 were considered statistically significant.

### Supplementary information

Original Data

## Data Availability

All data are available in the main text or the supplementary materials.
