# Supplementary material for: N-ethylmaleimide-sensitive factor elicits a neuroprotection against ischemic neuronal injury by restoring autophagic/lysosomal dysfunction
Source: Cell Death Discov. 2024 Aug 18;10:368. doi: 10.1038/s41420-024-02144-7 (PMC11330971; doi:10.1038/s41420-024-02144-7)
Supplement: Supplementary file 1 — Original Data [file 41420_2024_2144_MOESM1_ESM.docx]

**Original image: Figure 1**

**D7**

**D6**

**D5**

**D4**

**D3**

**D2**

**D1**

**H12**

**H6**

**H5**

**H4**

**H3**

**H2**

**H1**

**Sham**

**
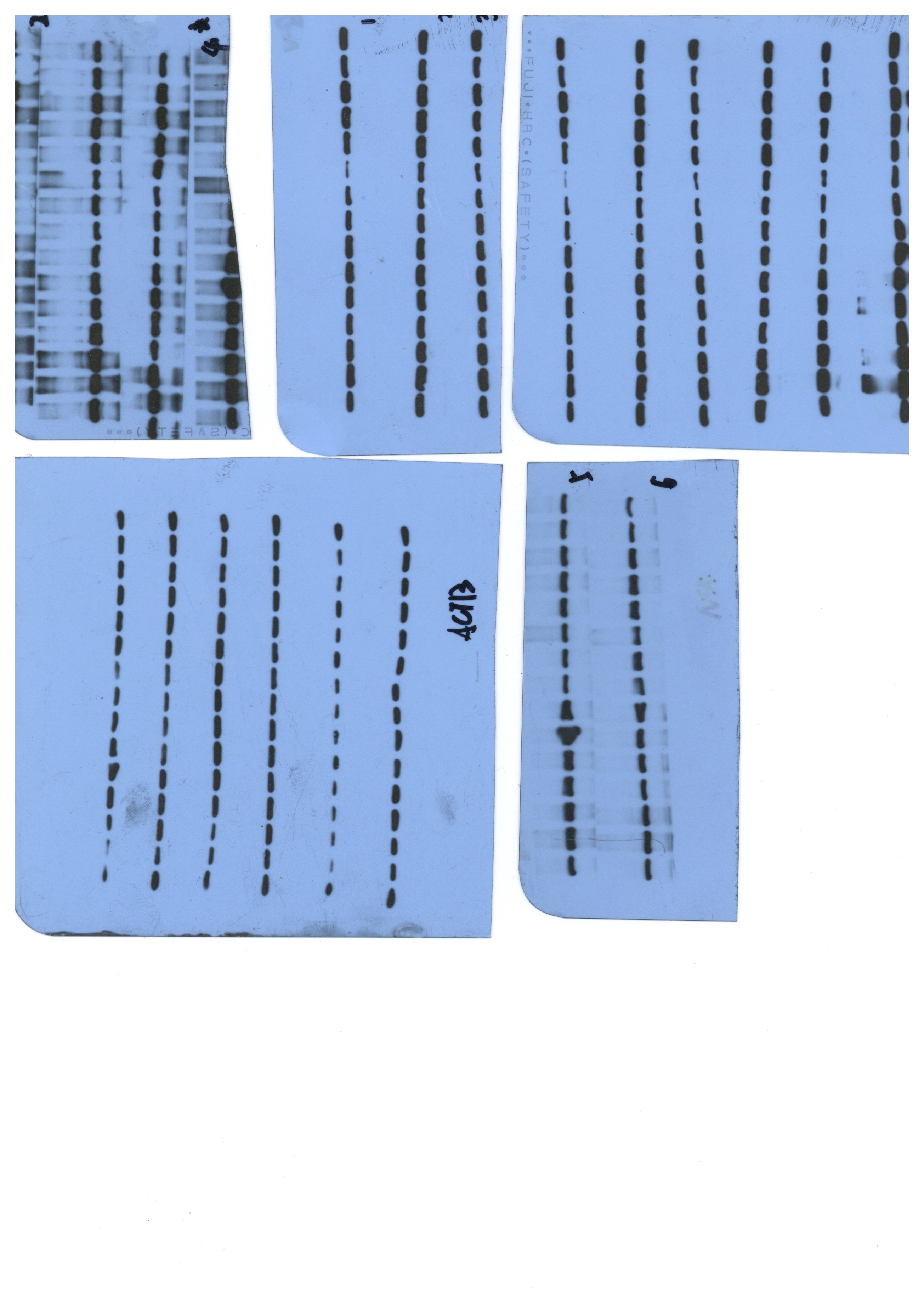
**

**NSF**

**D7**

**D6**

**D5**

**D4**

**D3**

**D2**

**D1**

**H12**

**H6**

**H5**

**H3**

**H4**

**H1**

**H2**

**Sham**

**
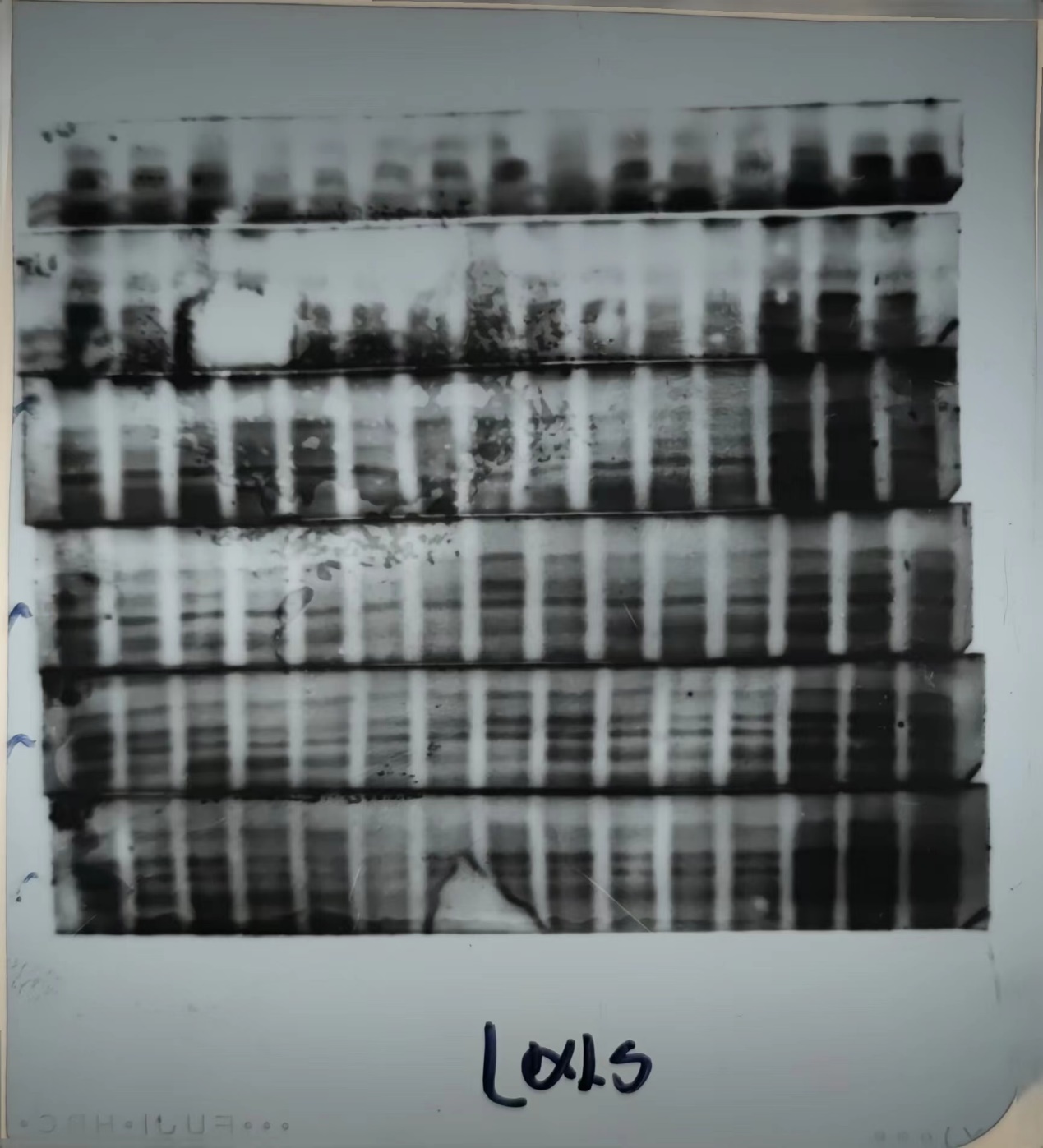
**

**STX17**

**H12**

**H6**

**H5**

**H4**

**H3**

**H2**

**H1**

**Sham**

**D1**

**D4**

**D2**

**D3**

**D5**

**D6**

**D7**

**
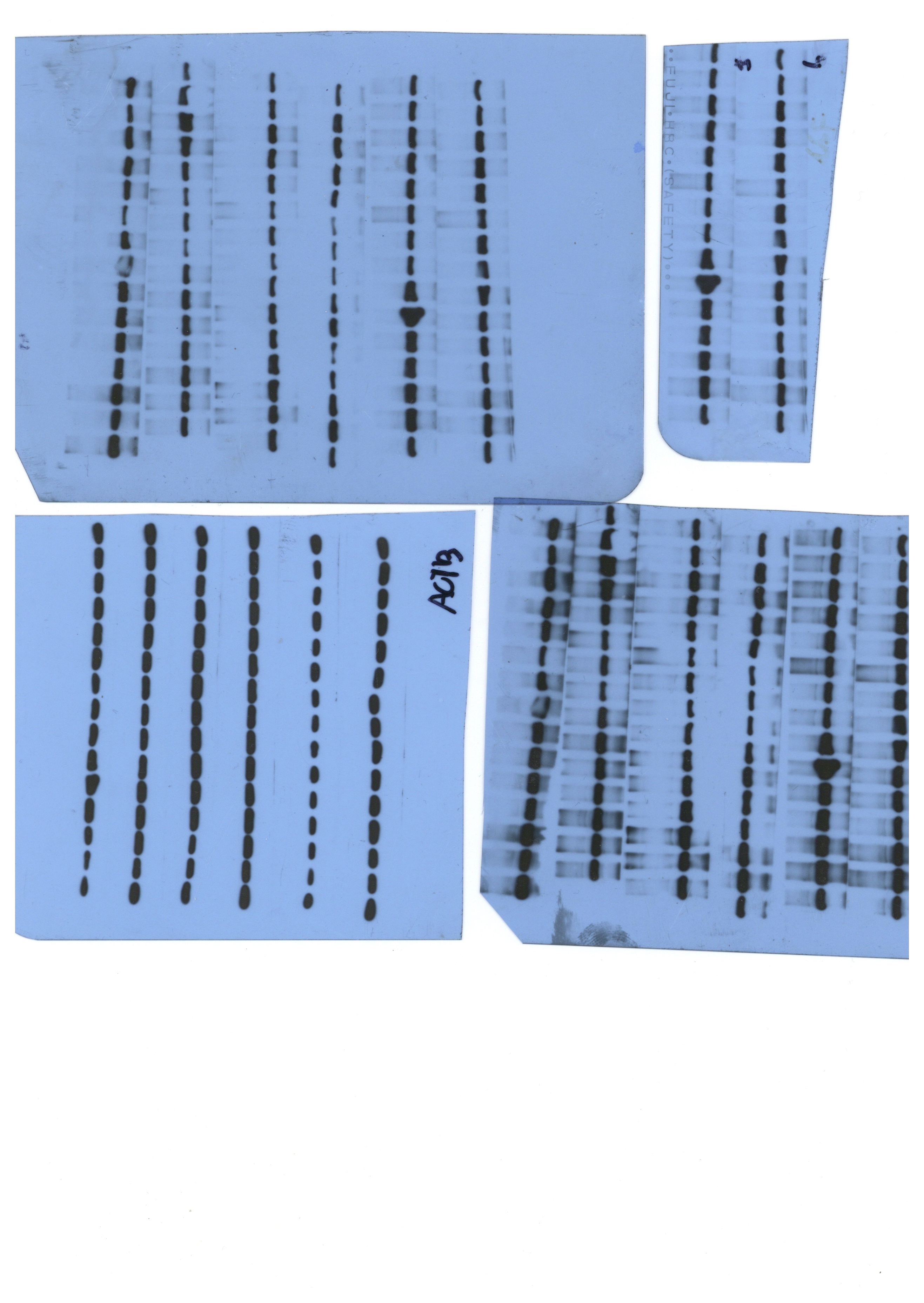
**

**VAMP8**

**D5**

**D4**

**D3**

**D2**

**D1**

**H12**

**D6**

**D7**

**H6**

**H5**

**H4**

**H3**

**H2**

**H1**

**Sham**

**
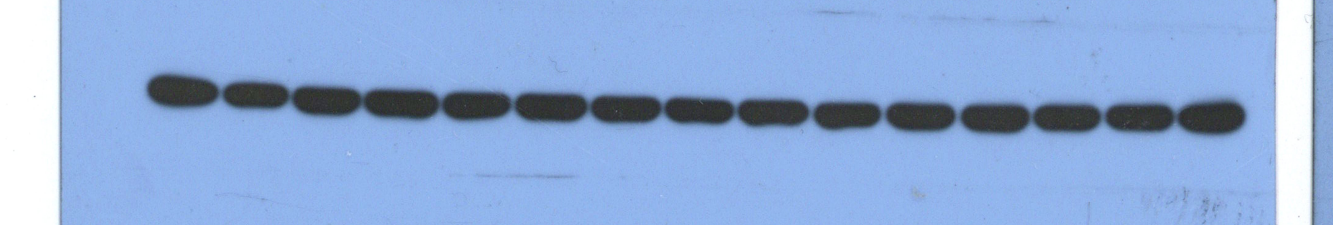
**

**ACTB**

**Original image: Figure 2A**

**Sham**

**D2**

**Beclin-1**

**
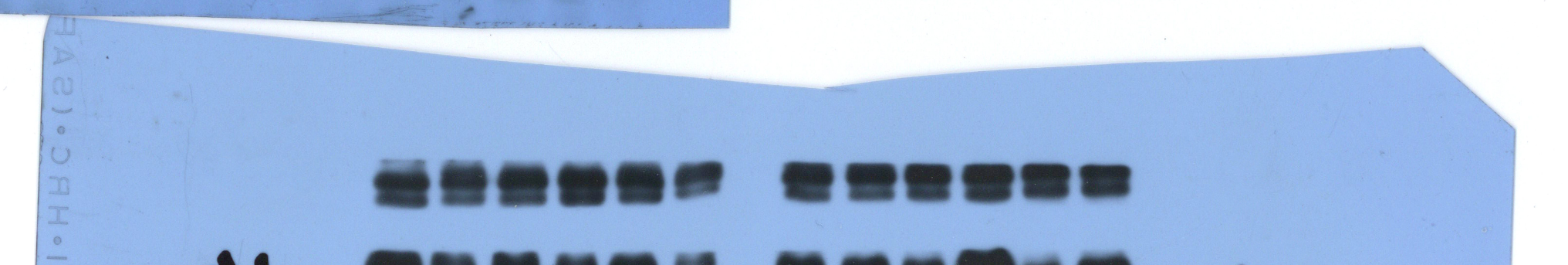
**

**
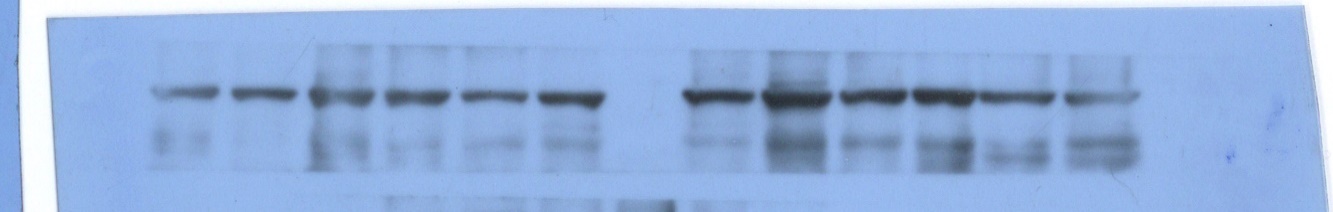
**

**LAMP-2**

**
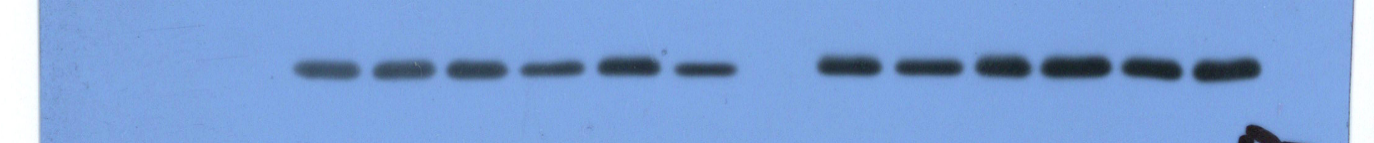
**

**ACTB in the artical**

**
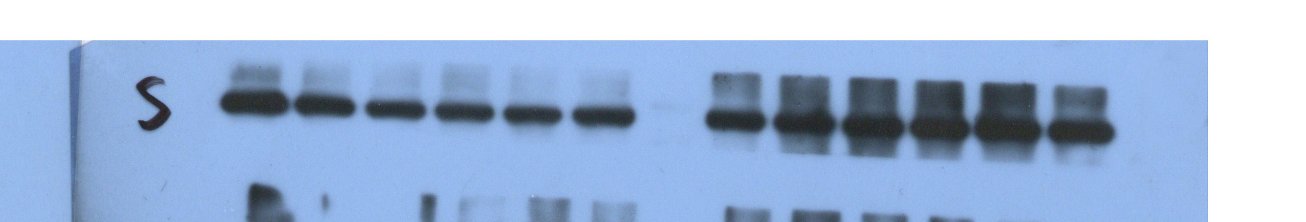
**

**Soluble SQSTM1**

**
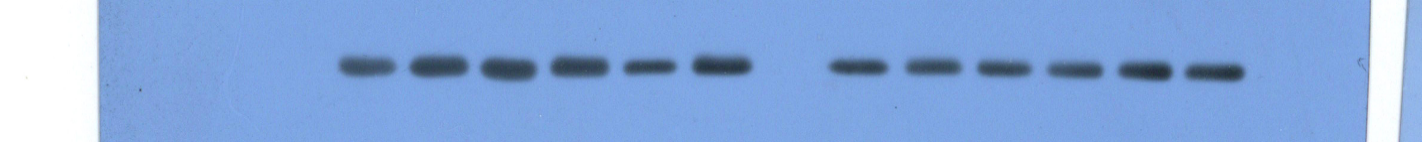
**

**ACTB**

**
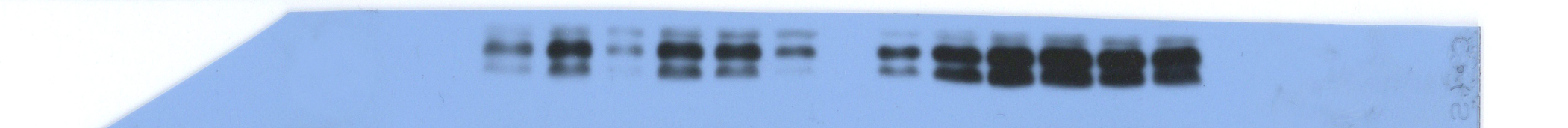
**

**Insoluble SQSTM1**

**
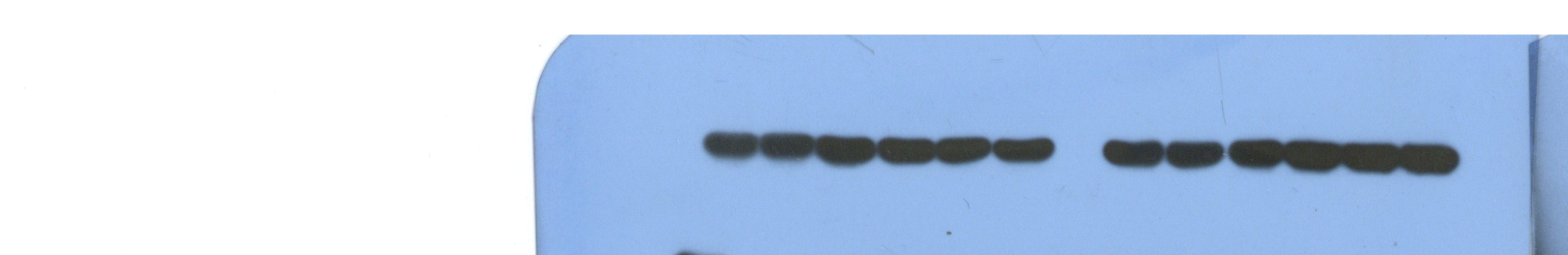
**

**ACTB**

**D2**

**Sham**

**Pro-CTSD**

**
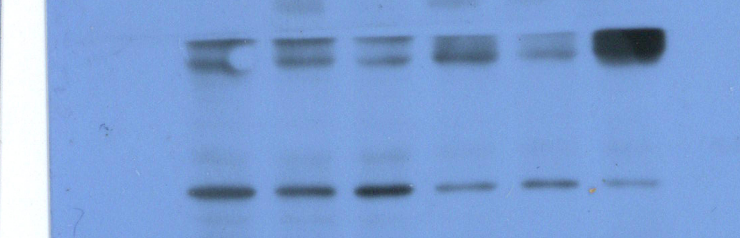
**

**Mat-CTSD**

**
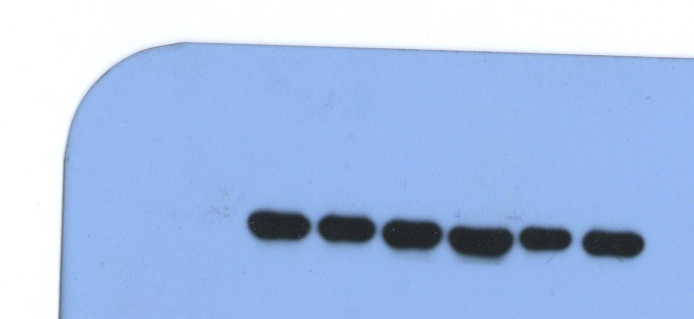
**

**ACTB**

**Sham**

**D2**

**
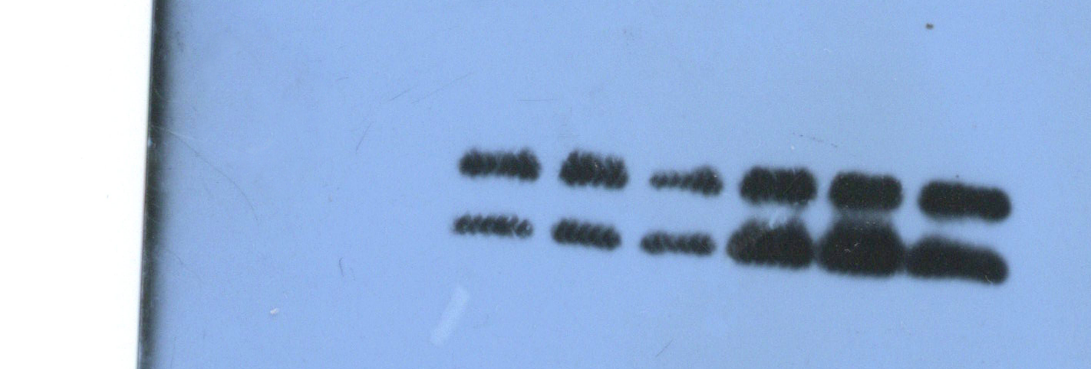
**

**LC3-Ⅰ**

**LC3-Ⅱ**

**
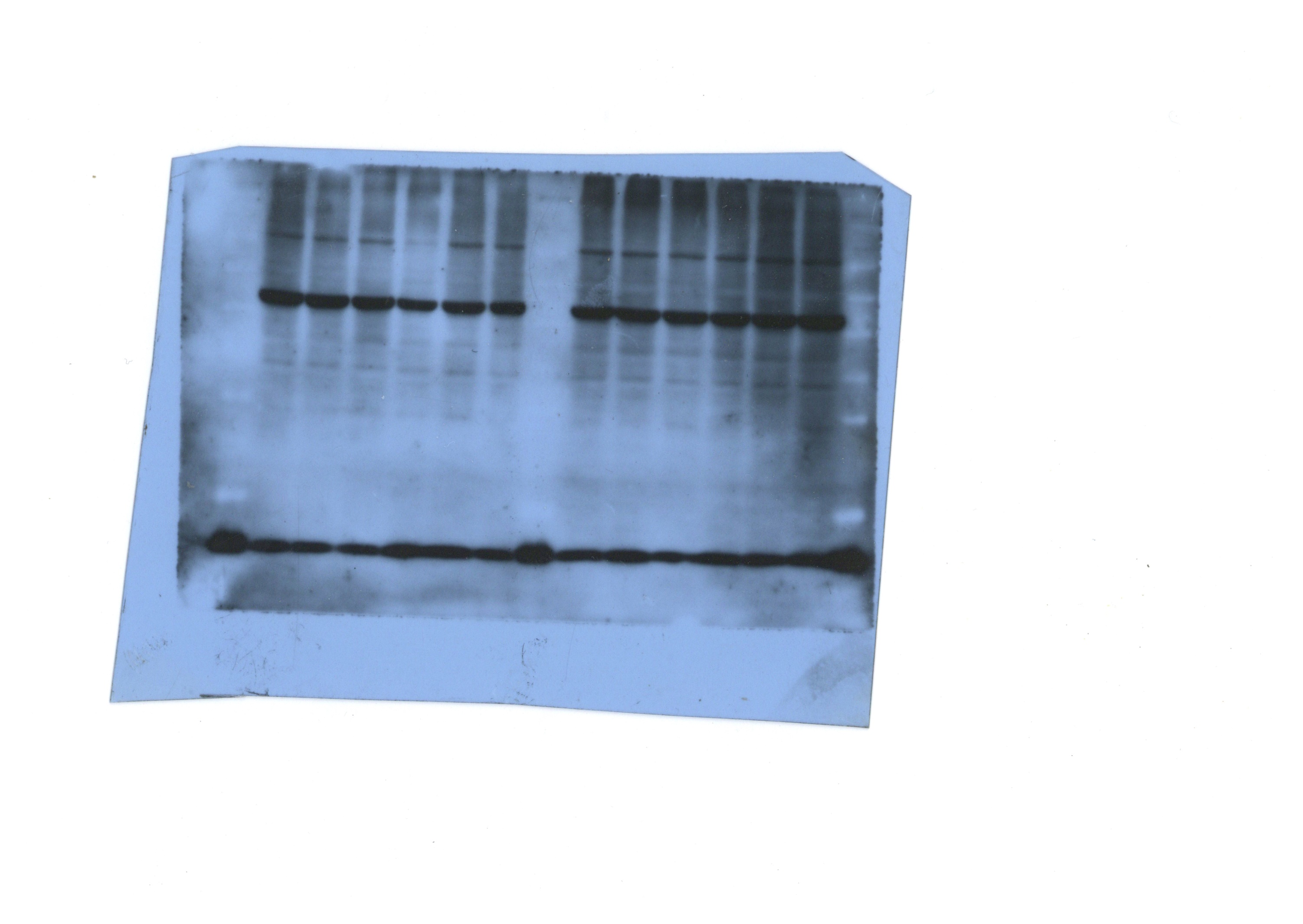
**

**Ubiquitin**

**
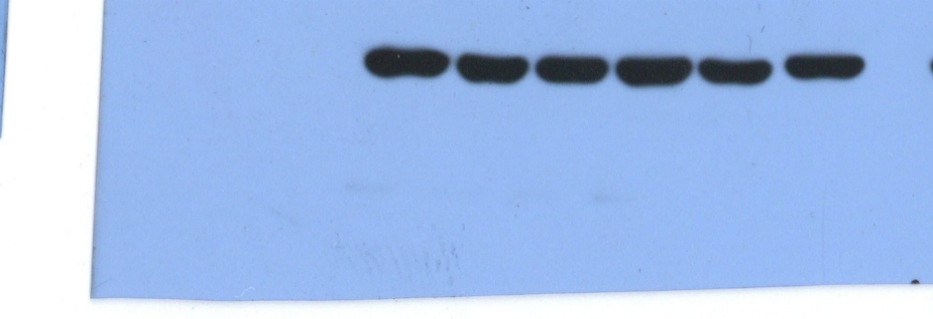
**

**ACTB**

**Original image: Figure 3A**

**Ctrl**

**OGD**

**
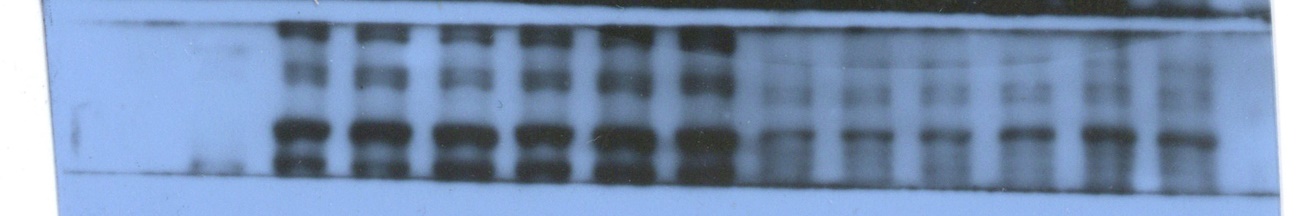
**

**NSF**

**
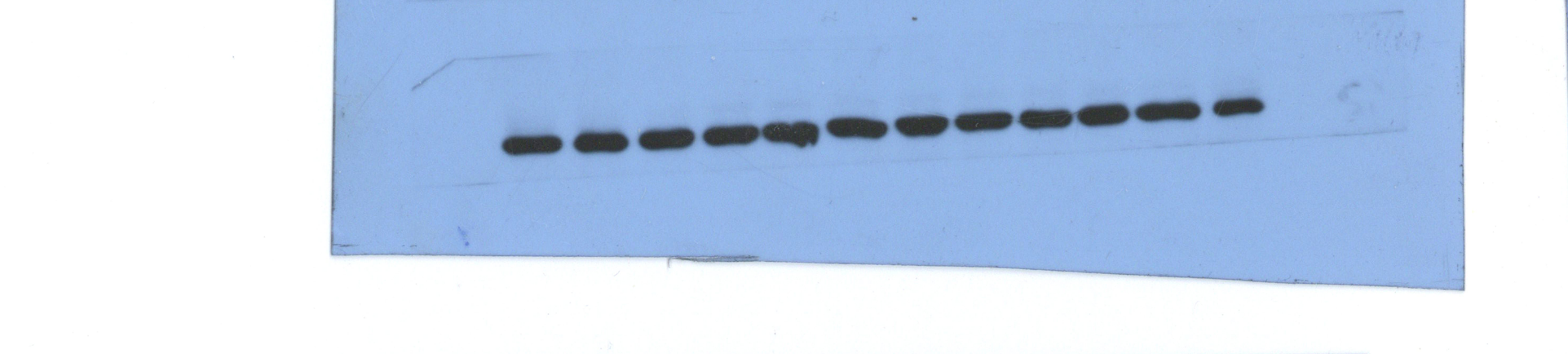
**

**ACTB**

**Original image: Figure 3E**

**si-NSF**

**si-NC**

**Lv-NSF**

**Lv-Ctrl**

**OGD**

**Ctrl**


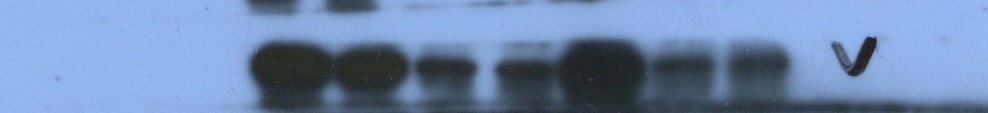


**NSF**

**
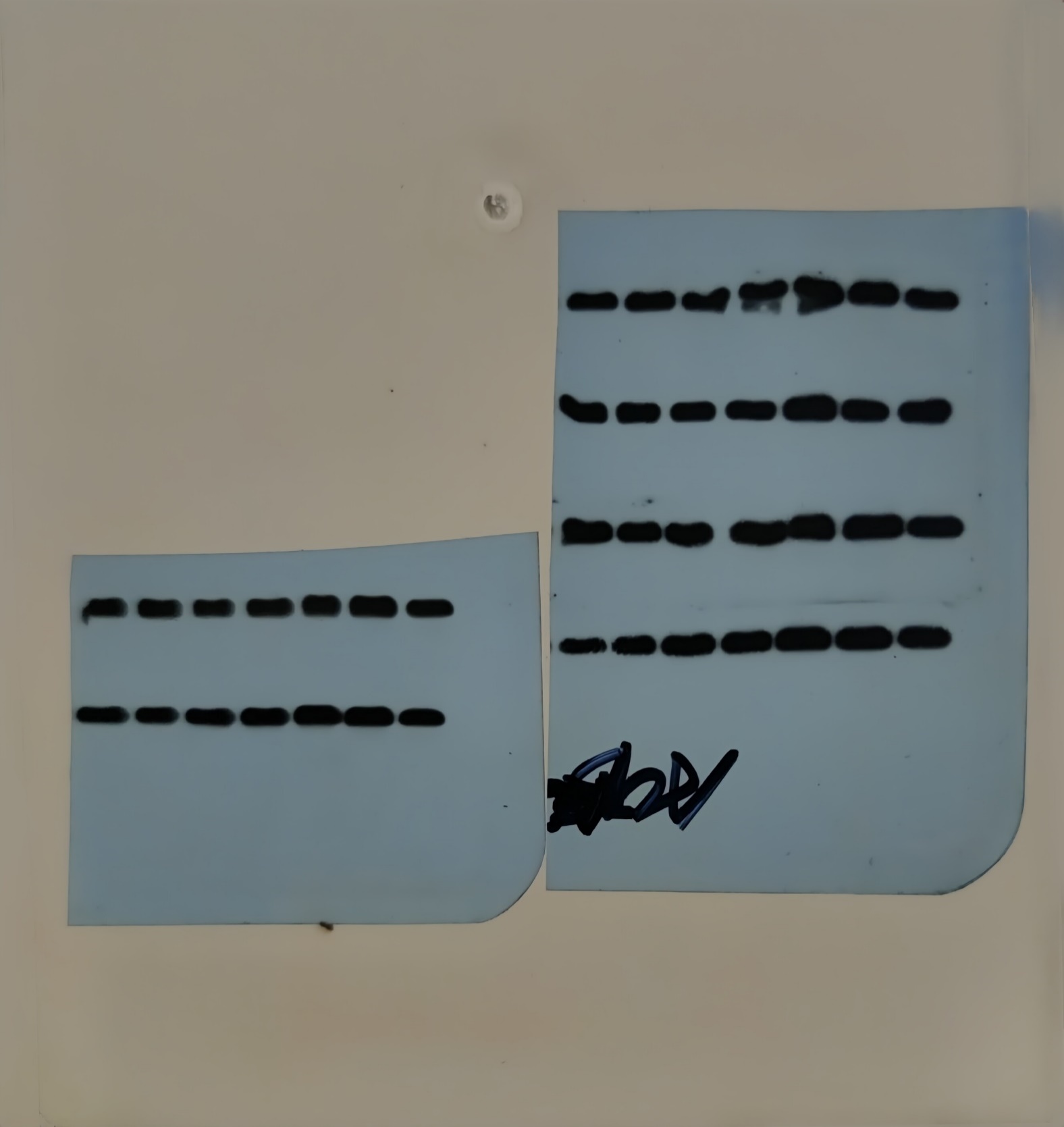
**

**ACTB**

**
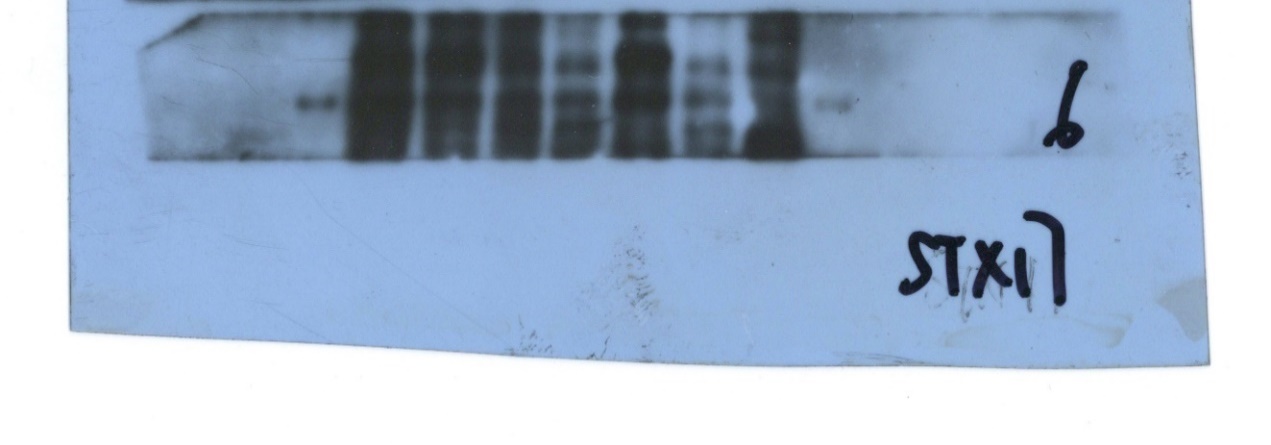

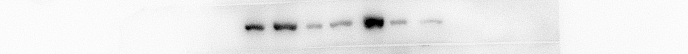
**

**STX17**

**VAMP8**

**
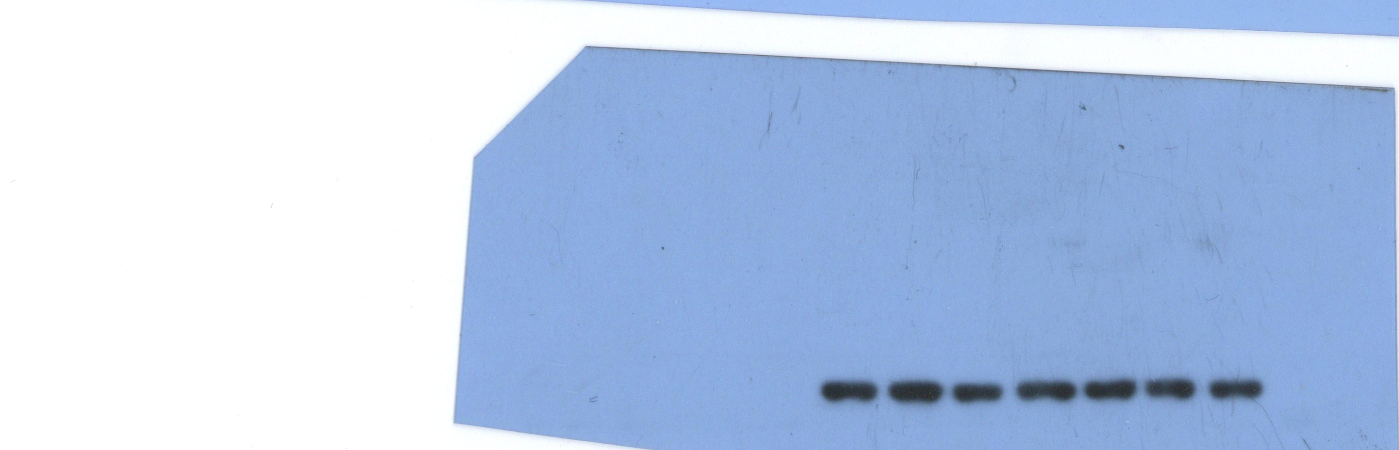
**

**ACTB in the artical**

**Original image: Figure 4A**

**Ctrl**

**OGD**

**Lv-Ctrl**

**Lv-NSF**

**si-NC**

**si-NSF**

**IgG**

**
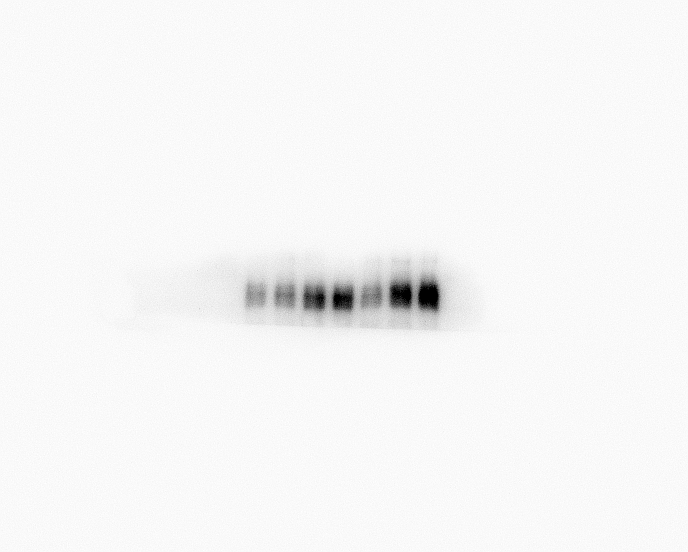
**

**STX17**

**
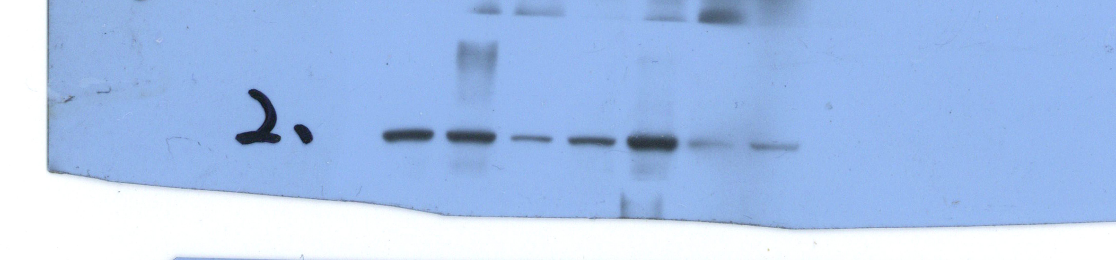
**

**VAMP8**

**Original image: Figure 4B**

**Ctrl**

**OGD**

**Lv-Ctrl**

**Lv-NSF**

**si-NSF**

**si-NC**

**
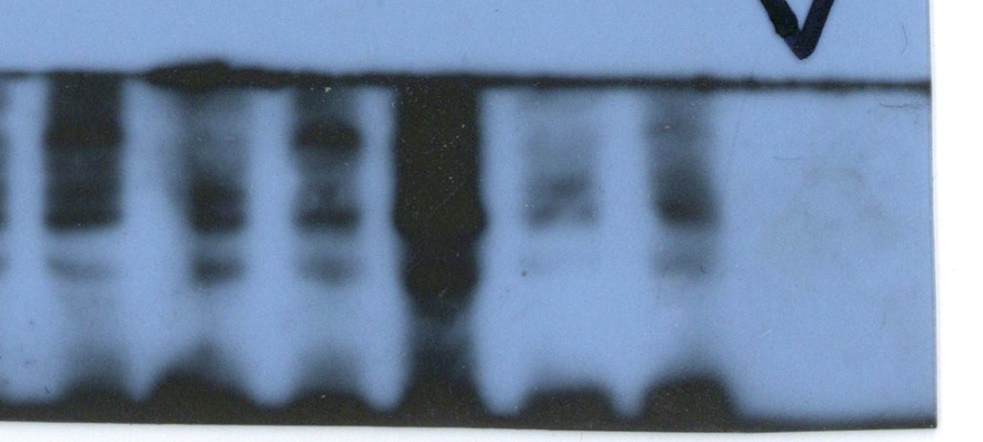
**

**STX17**

**
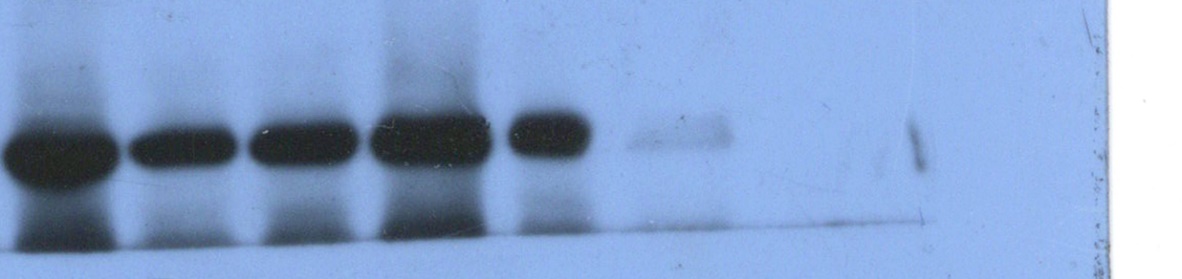
**

**VAMP8**

**
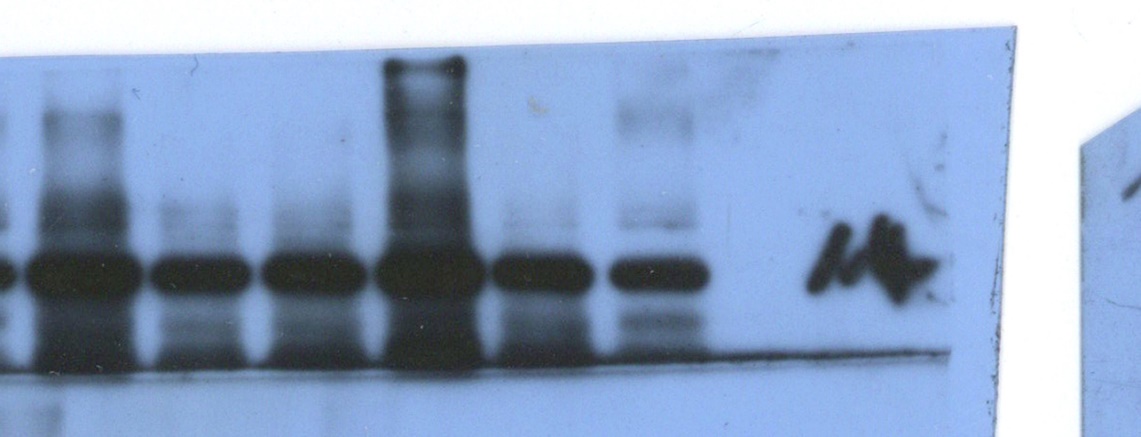
**

**NSF**

**
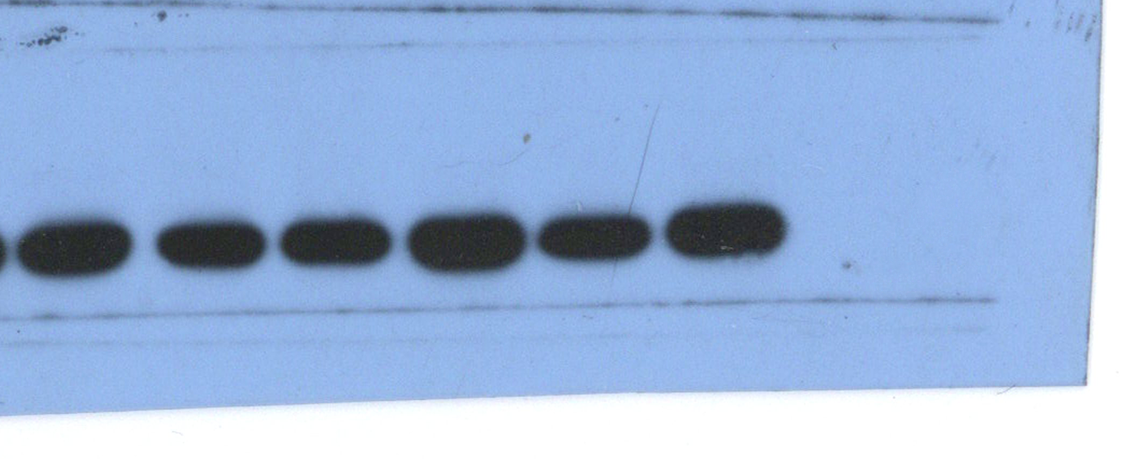
**

**ACTB**

**Original image: Figure 6A**

**si-NSF**

**Ctrl**

**OGD**

**Lv-Ctrl**

**Lv-NSF**

**si-NC**

**
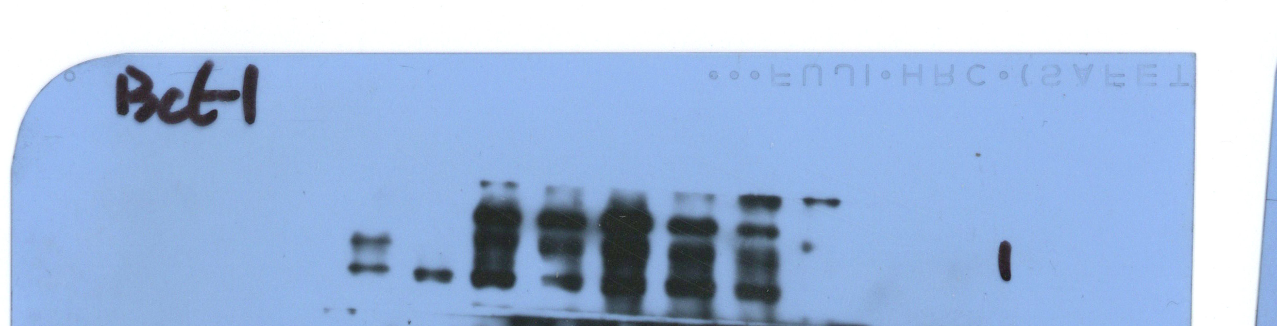
**

**Beclin-1**

**
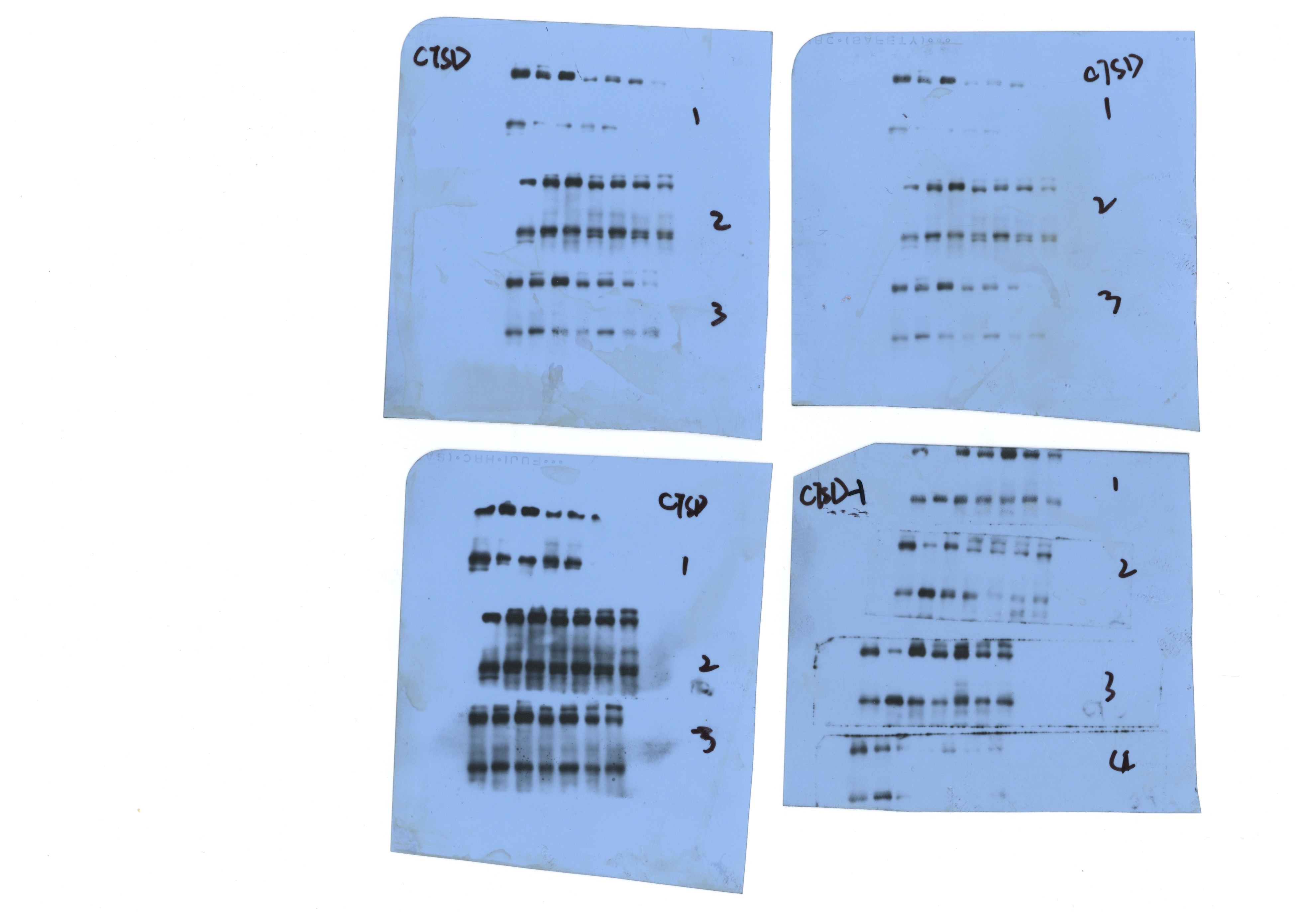
**

**Pro-CTSD**

**Mat-CTSD**

**
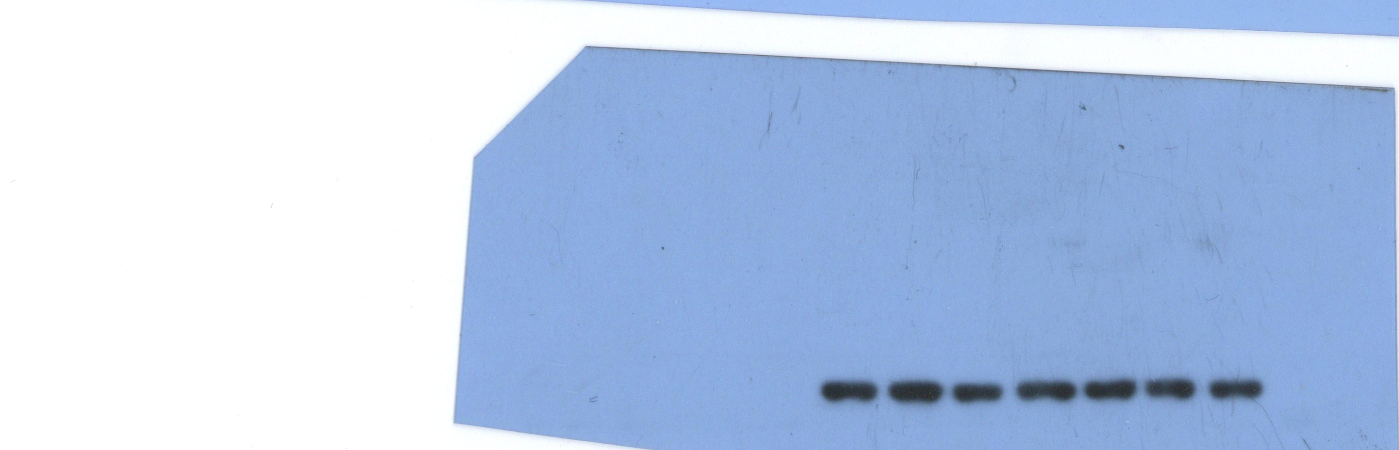
**

**ACTB**

**si-NSF**

**si-NC**

**Lv-NSF**

**Lv-Ctrl**

**OGD**

**Ctrl**

**
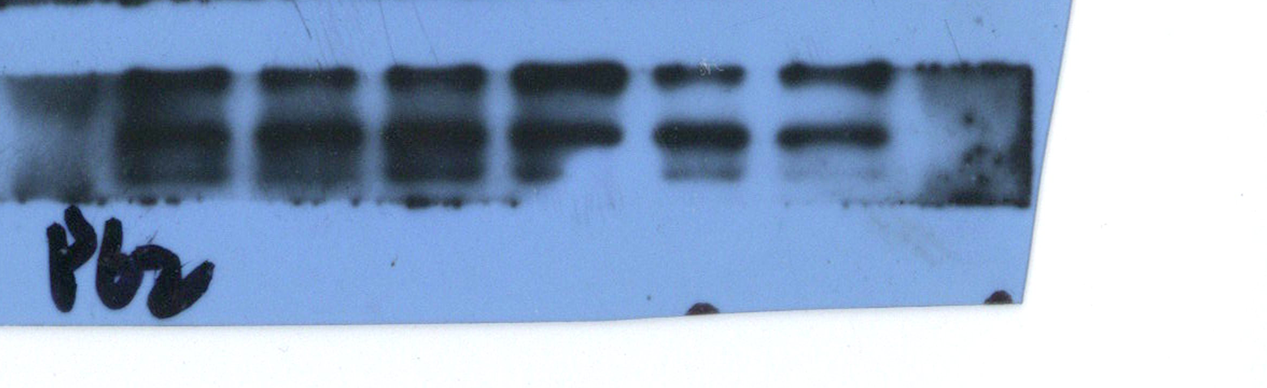
**

**Soluble SQSTM1**

**
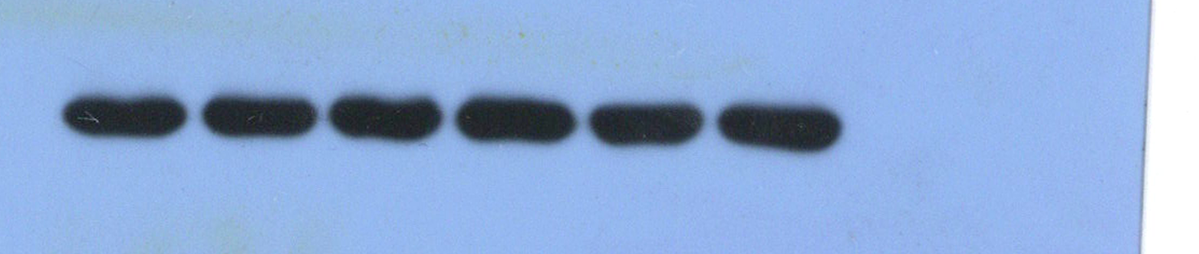
**

**ACTB in the artical**


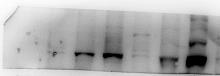


**Insoluble SQSTM1**

**ACTB**

**
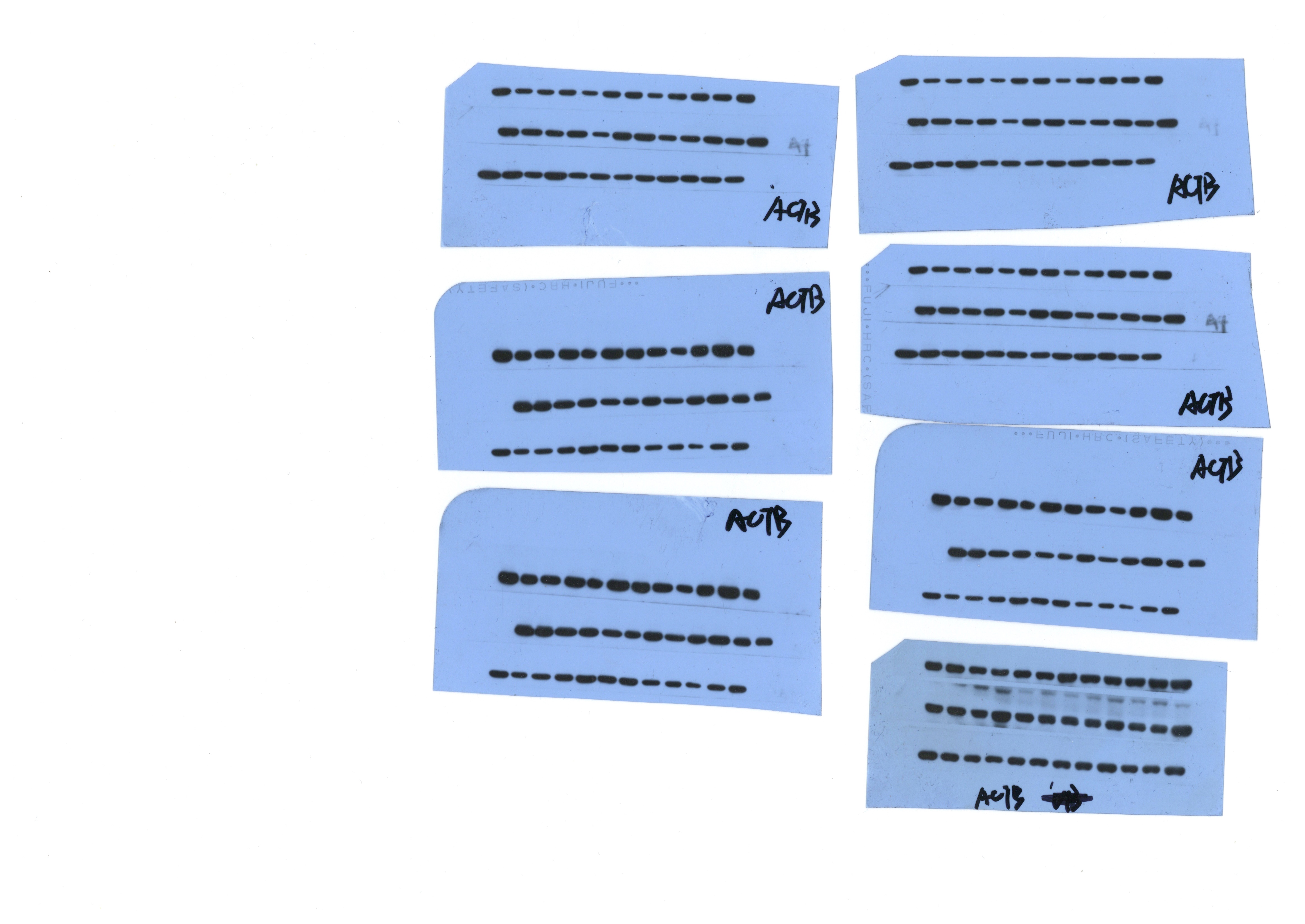
**

**
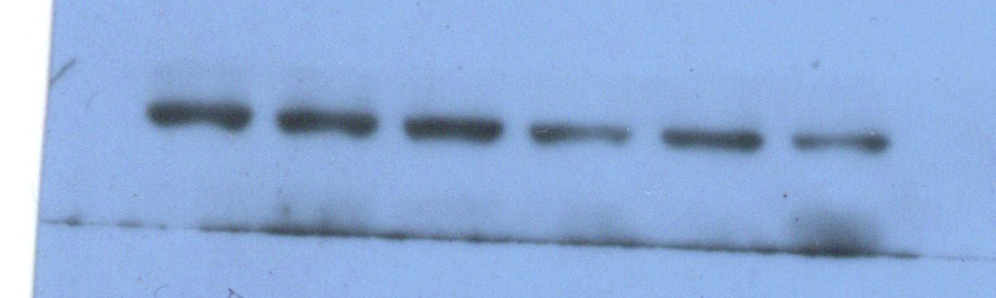
**

**LAMP-2**


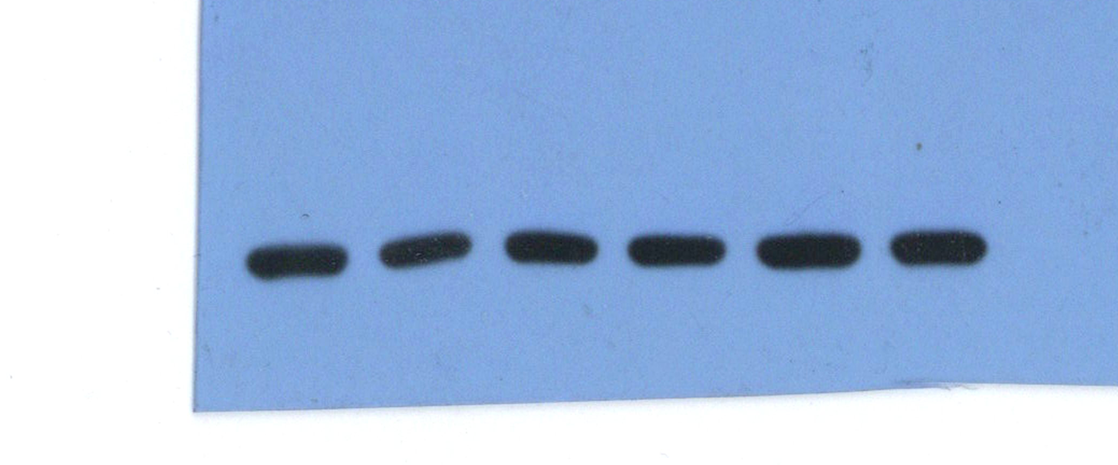


**ACTB**

**
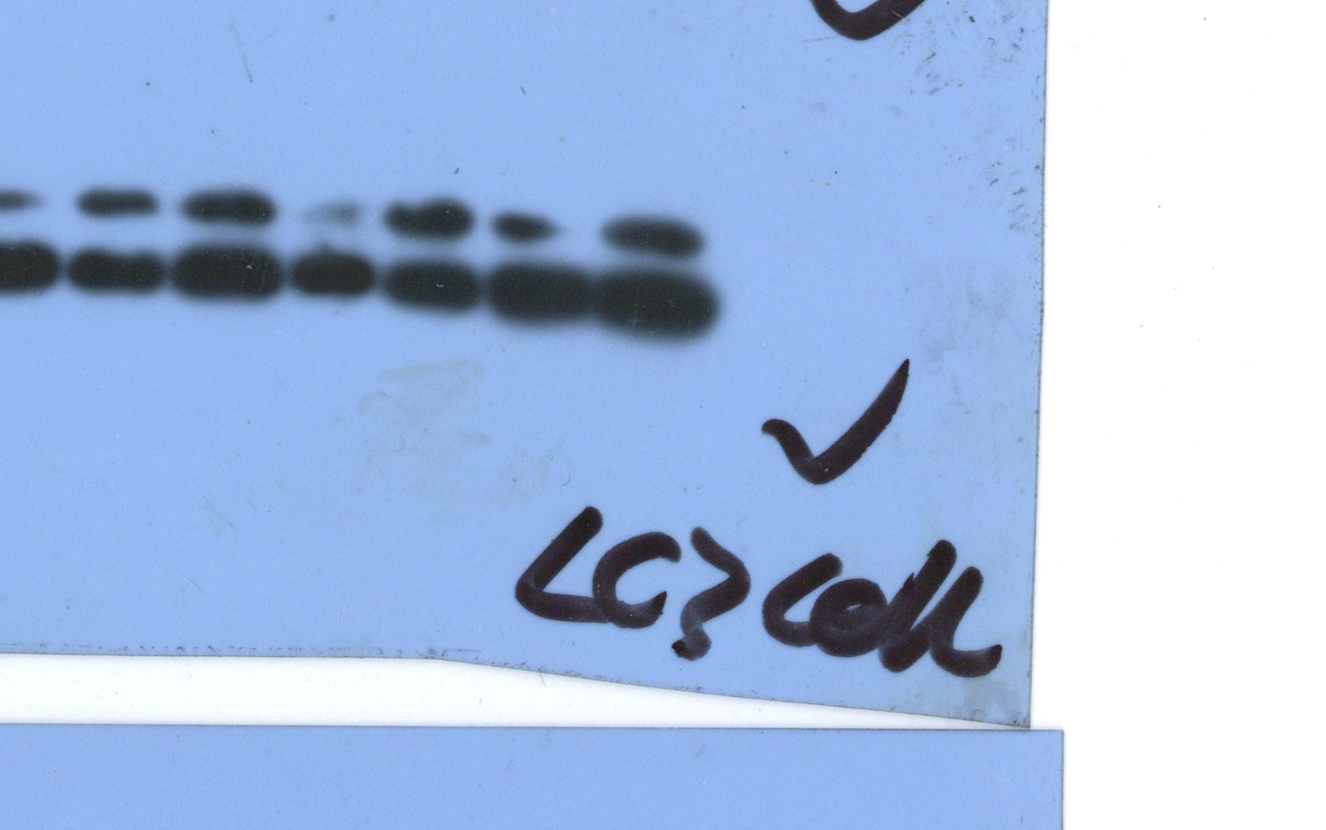
**

**LC3-Ⅱ**

**LC3-Ⅰ**

**
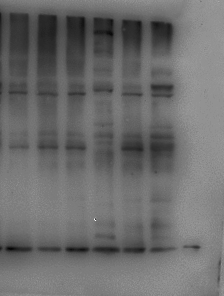
**

**Ubiquitin**

**
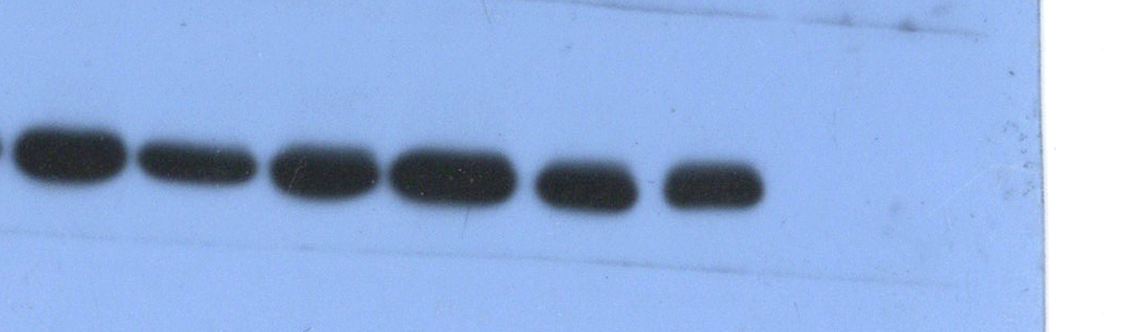
**

**ACTB**
